# Supplementary material for: Inequalities in the care experiences of patients with cancer: analysis of data from the National Cancer Patient Experience Survey 2011–2012
Source: BMJ Open. 2014 Feb 14;4(2):e004567. doi: 10.1136/bmjopen-2013-004567 (PMC3927926; doi:10.1136/bmjopen-2013-004567)
Supplement: Author's manuscript [file bmjopen-2013-004567.draft_revisions.pdf]

Inequalities in cancer patients' experiences of care: analysis of data from the National Cancer Patient Experience Survey 2011-12

Journal:	*BMJ Open*
Manuscript ID:	bmjopen-2013-004567
Article Type:	Research
Date Submitted by the Author:	27-Nov-2013
Complete List of Authors:	Bone, Anna; Imperial College London, School of Public Health<br>Mc Grath-Lone, Louise; Imperial College London, Patient Experience Research Centre, School of Public Health<br>Day, Sophie; Imperial College London, Patient Experience Research Centre, School of Public Health<br>Ward, Helen; Imperial College London, Patient Experience Research Centre, School of Public Health
**Primary Subject Heading**:	Public health
Secondary Subject Heading:	Oncology
Keywords:	PUBLIC HEALTH, ONCOLOGY, Adult oncology < ONCOLOGY

SCHOLARONE™
Manuscripts

Inequalities in cancer patients' experiences of care: analysis of data from the National Cancer Patient Experience Survey 2011-12

Corresponding author: Louise Mc Grath-Lone, Patient Experience Research Centre, School of Public Health, Imperial College London, W2 1PG, UK.
E-mail:Louise.mc-grath-lone@imperial.ac.uk
Phone: 0207 5943417 Fax: 0207 4023927

Authors: Anna Bone[1], Louise Mc Grath-Lone[2], Sophie Day[2], Helen Ward.[2]

Affiliations:

1. School of Public Health, Imperial College London, W2 1PG, UK.
2. Patient Experience Research Centre, School of Public Health, Imperial College, London, W2 1PG, UK.

Keywords: Patient satisfaction, quality measurement, surveys

Word count: 2,814

For peer review only - http://bmjopen.bmj.com/site/about/guidelines.xhtml

ABSTRACT

Background

Patient experience is a key component of quality in healthcare; however, studies in the UK indicate that patients' experience of care is not equal across gender, age and ethnicity. There are currently 1.8 million people living with cancer in the UK but less is known about their experiences of care. Whether inequalities by socio-demographic characteristics exist amongst cancer patients is not well-documented.

Methods

Using cross-sectional data from 71,793 cancer patients who completed the National Cancer Patient Experience Survey 2011-2012, we examined associations between patient, clinical and trust-level factors and a summary measure of patient experience, namely overall rating of care. Multivariate logistic regression was used to investigate variation by socio-demographic characteristics adjusting for other patient, clinical and trust-level factors.

Results

Female, non-white and younger patients were less likely to rate their overall care as excellent or very good. Patients with long-standing conditions, particularly those with learning disabilities or mental health conditions also reported poorer overall care. This variation persisted when other patient, clinical and trust-level factors were controlled

For peer review only

For peer review only - http://bmjopen.bmj.com/site/about/guidelines.xhtml

for, indicating there are real differences in experiences among cancer patients by socio-demographic characteristics.

Conclusion

There is evidence of inequalities in cancer patient experience in the UK by socio-demographic characteristics such as gender, age, ethnicity and disability. Quality cancer care services must strive to meet the needs of a diverse patient population equally; this study identifies patient groups for whom it appears cancer care services are in greatest need of improvement.

Article summary: Strengths and limitations of this study

- To the authors' knowledge, this is the first study to explore cancer patients' overall rating of care by socio-demographic characteristics including longstanding-conditions or disabilities.
- A principal strength of this study is the large sample size (> 71,000) and high response rate (68%).
- As this study involves secondary analysis of national survey data it is limited by the type of data available e.g. the influence of potentially important predictors of patient experience such as employment status, level of deprivation and health status could not be explored as these data were not gathered.
- A further limitation is that the binary categorisation of patients' responses condenses the patients' experiences (which were already limited to several multiple choice options) and may mask potentially significant variation.

For peer review only - http://bmjopen.bmj.com/site/about/guidelines.xhtml

INTRODUCTION

Patient experience is a key component of quality in healthcare and is one of the top priorities in the NHS (1). However, studies in the UK indicate that there are systematic differences in patient experience by socio-demographic characteristics (2–4). For example, studies on primary and hospital care have found that patients tend to report more positive experiences with increasing age (4–8), females report less positive experiences than males (5,6) and non-white patients report less positive experiences than white patients, even after adjusting for other socio-demographic variables (3,5,6,9). Less is known about variation in the experiences of cancer patients. There are currently 1.8 million people living with cancer in the UK (10) and advances in cancer treatments mean that they are living longer and facing prolonged periods of contact with healthcare services because of complex treatment regimens (11). In 2011, a Department of Health (DH) report set out the government's strategy to improve outcomes by putting patients at the heart of cancer health services (10). A key objective of this strategy was to reduce inequalities in care relating to both clinical outcomes and patient experience. Thus, exploration of the experiences of cancer patients and the inequalities that may exist is critical in order to identify patient groups for whom cancer care services are in greatest need of improvement.

Detailed studies on the experiences of cancer patients have often relied on small sample sizes (12–14). However, a regular National Cancer Patient Experience Survey (NCPES) has been established which provides a wealth of information on care and treatment experiences. The 2011-12 survey includes responses from over 71,000 cancer patients from 160 trusts across the UK (15). With a relatively high response rate and large sample size, the survey presents an opportunity to explore inequalities

For peer review only - http://bmjopen.bmj.com/site/about/guidelines.xhtml

For peer review only

in the quality of care received by cancer patients. Here, we aim to describe the variation in cancer patient experience by age, gender, ethnicity and presence of long-standing conditions or disabilities in order to explore whether there are systematic inequalities. We further examine the influence of clinical and trust-level factors on these variations.

METHODS

Source of data

We carried out a secondary analysis of 2011-2012 NCPES cross-sectional data collected by Quality Health (Chesterfield, UK) on behalf of the DH. All patients with a primary diagnosis of cancer who attended an NHS hospital as an inpatient or day case between 1st September 2011 and 30th November 2011 were sent the survey (15). Non-responders were followed up with two postal reminders. The final response rate achieved was 68%. As no survey weights were available, the data could not be weighted to adjust for non-response. The dataset included demographic and clinical characteristics for 71,793 cancer patients who attended 160 hospital trusts across England, as well as their responses to 70 multiple choice questions relating to various aspects of their experiences of care. Surveys such as NCPES are commonly used to measure patient experience over a range of domains; however, single summary measures of overall experience, such as the Family and Friends Test, have become increasingly important (1). Our analysis focused on a summary measure of patient experience, namely patients' assessment of care as measured by Q70 in the survey, *"Overall, how would you rate your care?"* Responses from a five point scale were transformed into a binary outcome, with "*excellent*" and "*very good*" categorised as

For peer review only - http://bmjopen.bmj.com/site/about/guidelines.xhtml

'positive' and *"good"*, *"fair"* and *"poor"* as 'not positive', in accordance with the DH Survey Guidance (15).

Patient, clinical and trust-level characteristics

The main socio-demographic characteristics of interest in this study (gender, age and ethnicity) were ascertained by self-report (16) and grouped as in the national report (15). As Chinese patients have reported less positive experiences than white patients elsewhere (3,6,17), the "Chinese" ethnic category was not combined with "Other" in this analysis. For age and ethnicity the largest groups were chosen as the reference category. Responses to the question "*Do you have any of the following long-standing conditions?"* were used to identify patients with co-morbidities. The clinical characteristics of tumour group and patient status (i.e. day or inpatient) were taken from hospital administration records.

Haematological cancer patients were assigned as the reference tumour group as the largest group (breast) did not have a representative age and gender distribution. Time since first treatment was ascertained by patients' survey responses. As trust-level factors have previously been associated with patient experience (6,18–20) several were included in this analysis. Hospital trusts were categorised by type (large acute, medium acute, small acute, specialist and teaching) and by foundation status. The Care Quality Commission's (CQCs) 2008/2009 Annual Health Check rating and the proportion of frontline staff satisfied with care at their trust (Q12d from the National NHS Staff Survey 2012) were also included as measures of trusts' overall quality. Quintiles of staff satisfaction were used as a categorical variable during regression

For peer review only - http://bmjopen.bmj.com/site/about/guidelines.xhtml

analysis with the lowest quintile as reference category. The reference categories chosen for other trust-level factors were the largest groups.

Data analysis

Variation in patents' overall rating of care by patient, clinical and trust-level factors was investigated using univariate logistic regression. Multivariate logistic regression was then used to describe associations between the individual demographic characteristics of interest and overall rating of care. Confounding by patient, clinical and trust-level factors was controlled for through their sequential addition to the model. Logistic regression was chosen as the small intra-class coefficient calculated for Q70 (<0.01) suggested the effect of clustering by trust among respondents was negligible; therefore, it was anticipated that a multilevel model and a multivariate logistic regression model would produce similar results (21). However, as even small intra-class correlations can inflate type-1 errors, clustered robust standard errors were utilised. Respondents with missing demographic, clinical or trust-level data, or those who did not answer Q70, were excluded (i.e. complete-case analysis was undertaken). All statistical analyses were conducted in Stata V.12.

For peer review only

For peer review only - http://bmjopen.bmj.com/site/about/guidelines.xhtml

RESULTS

Patient characteristics and rating of care

A total of 71,793 patients admitted to hospital trusts across England with a primary cancer diagnosis completed the survey. Table 1 shows the demographic and clinical characteristics of respondents and Table 2 the characteristics of the hospital trusts they attended. The majority of patients were white, female and >50 years old and there were substantial numbers with disabilities or other long-standing conditions. The most common tumour groups were breast and haematological cancers. Most respondents had started their treatment in the last year and were admitted to hospital as day case patients on their most recent visit. Most were treated in large acute trusts, trusts with foundation status and trusts rated 'Good' by CQC. The majority of patients (96.5%, n=69,276) provided a response to Q70 "*Overall, how would you rate your care?*" In total 87.8% rated their care as "*excellent*" or "*very good*" while the remaining 12.2% rated their care "*good"*, "*fair*" or "*poor*" (Fig.1).

Figure 1: Responses from NCPES 2011-12 to Q70
"Overall, how would you rate your care?"

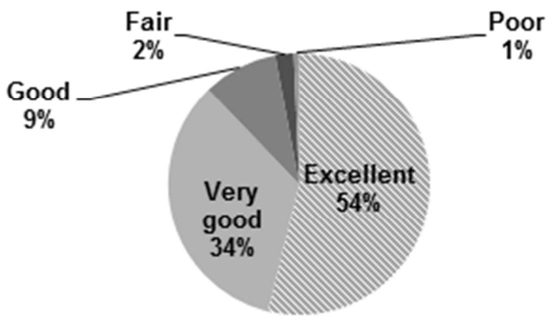

For peer review only - http://bmjopen.bmj.com/site/about/guidelines.xhtml

Table 1: Characteristics of survey respondents and their unadjusted associations with a positive overall rating of care from univariate logistic regression

Patient characteristics

**Gender**	**n**	**%**	**OR (95% CI)**	***p*-value**
Male	33,832	47.1	1 (ref)	
Female	37,961	52.9	0.92 (0.88-0.96)	<0.001*
**Age group**	**n**	**%**	**OR (95% CI)**	***p*-value**
16 - 25	363	0.5	0.68 (0.51-0.92)	0.01*
26 - 35	969	1.4	0.62 (0.52-0.75)	<0.001*
36 - 50	6,802	9.5	0.70 (0.64-0.76)	<0.001*
51 - 65	22,885	31.9	0.79 (0.74-0.83)	<0.001*
66 - 75	23,643	32.9	1 (ref)	
76+	17,131	23.9	0.85 (0.79-0.90)	<0.001*
**Ethnicity**	**n**	**%**	**OR (95% CI)**	***p*-value**
White	63,652	88.7	1 (ref)	
Mixed	199	0.3	0.66 (0.45-0.97)	0.04*
Asian/Asian British	1,082	1.5	0.33 (0.29-0.38)	<0.001*
Black/Black British	885	1.2	0.41 (0.35-0.49)	<0.001*
Chinese	138	0.2	0.27 (0.19-0.39)	<0.001*
Other	510	0.7	0.58 (0.46-0.73)	<0.001*
**Long-standing conditions** [a, b]	**n**	**%**	**OR (95% CI)**	***p*-value**
None	48,218	67.2		
Deafness/hearing impairment	7,281	10.1	0.91 (0.85-0.98)	0.01*
Blindness/partially sighted	1,856	2.6	0.74 (0.65-0.84)	<0.001*
Physical condition	9,347	13.0	0.71 (0.67-0.76)	<0.001*
Learning disability	354	0.5	0.50 (0.39-0.65)	<0.001*
Mental health condition	1,347	1.9	0.55 (0.48-0.64)	<0.001*
Long-standing illness [c]	9,241	12.9	0.77 (0.73-0.82)	<0.001*

Clinical characteristics

**Tumour group**	**n**	**%**	**OR (95% CI)**	***p*-value**
Brain/CNS	746	1.0	0.52 (0.42-0.63)	<0.001*
Breast	14,739	20.5	1.00 (0.92-1.09)	0.98
Colorectal / Lower GI	9,483	13.2	0.72 (0.66-0.78)	<0.001*
Gynaecological	4,202	5.9	0.72 (0.64-0.80)	<0.001*
Haematological	11,070	15.4	1 (ref)	
Head and Neck	2,422	3.4	0.71 (0.62-0.81)	<0.001*
Lung	5,029	7.0	0.77 (0.69-0.85)	<0.001*
Other	1,138	1.6	0.72 (0.59-0.86)	<0.001*
Prostate	5,831	8.1	0.70 (0.64-0.78)	<0.001*
Sarcoma	2,451	3.4	0.61 (0.53-0.69)	<0.001*
Skin	1,695	2.4	0.98 (0.82-1.16)	0.80
Upper GI	4,540	6.3	0.61 (0.55-0.68)	<0.001*
Urological	8,447	11.8	0.64 (0.58-0.70)	<0.001*
**Patient status**	**n**	**%**	**OR (95% CI)**	***p*-value**
Day case	45,720	63.7	1 (ref)	
Inpatient	26,073	36.3	0.84 (0.80-0.88)	<0.001*
**Time since first treatment**	**n**	**%**	**OR (95% CI)**	***p*-value**
< 1 year	44,997	62.3	1 (ref)	
1-5 years	17,486	24.4	0.83 (0.78-0.87)	<0.001*
>5 years	6,212	8.7	0.88 (0.81-0.95)	0.002*

Total number of respondents=71,793. Ethnicity was unknown for 7.4% respondents, long-standing conditions status for 7.3% and time since first treatment for 4.3%.

[a] 6.7% of patients (n=4,780) had >1 long-standing condition, therefore the column total exceeds 100%
[b] Reference category for specific long-standing conditions is not having that condition
[c] Such as (but not limited to) HIV, diabetes, chronic heart disease or epilepsy
*Significant at α=0.05 level

For peer review only

For peer review only - http://bmjopen.bmj.com/site/about/guidelines.xhtml

Table 2: Characteristics of trusts attended by survey respondents and their unadjusted associations with a positive overall rating of care from univariate logistic regression

Trust-level characteristics				
**Trust type**	**n**	**%**	**OR (95% CI)**	***p*-value**
Small acute	6,240	8.7	1.23 (1.12-1.34)	<0.001*
Medium acute	16,677	23.2	1.07 (1.01-1.14)	0.02*
Large acute	25,850	36.0	1 (ref)	
Specialist	3,224	4.5	1.54 (1.36-1.76)	<0.001*
Teaching	19,802	27.6	1.03 (0.98-1.10)	0.24
**Foundation status**	**n**	**%**	**OR (95% CI)**	***p*-value**
No	31,798	44.3	0.82 (0.78-0.85)	<0.001*
Yes	39,995	55.7	1 (ref)	
**CQC trust quality rating (2008/9)**	**n**	**%**	**OR (95% CI)**	***p*-value**
Weak	3,926	5.6	0.85 (0.77-0.94)	0.001*
Fair	18,482	26.2	0.97 (0.92-1.03)	0.34
Good	28,425	40.3	1 ref	
Excellent	19,748	28.0	1.17 (1.10-1.24)	<0.001*
**Frontline staff satisfied with care** [a]		**Quintiles of frontline staff satisfied with care**[b]		
Mean	63.5%		**OR (95% CI)**	***p*-value**
Median	62.7%	1 (lowest)	1 (ref)	
Range	35.3 - 94.0%	2	1.10 (1.03-1.19)	0.01*
		3	1.11 (1.03-1.19)	0.004*
		4	1.17 (1.09-1.26)	<0.001*
		5 (highest)	1.35 (1.25-1.45)	<0.001*

Total number of respondents=71,793. CQC trust quality rating was unknown for 3 trusts (1.7% of respondents)and the proportion of frontline staff satisfied with care was unknown for 1 trust (1.7% of respondents)

a Calculated from responses to Q12d from the National NHS Staff Survey 2012
b Trusts were categorised into quintiles according to the proportion of staff responding positively to Q12d
*Significant at α=0.05 level

Abbreviations: CQC=Care Quality Commission

Variation in rating of care by patient, clinical and trust-level factors

Unadjusted associations between patient, clinical and trust-level characteristics and Q70 from univariate logistic regression analysis are shown in Tables 1 & 2. Statistically significant variation in overall rating of care by patient-level characteristics such as ethnicity, gender, age and long-standing conditions was observed. For example, women were less likely than men, and non-white patients were less likely than white patients, to rate their overall care very good or excellent. Chinese patients reported least favourably among non-white ethnic minorities. Younger and older patients were

For peer review only - http://bmjopen.bmj.com/site/about/guidelines.xhtml

less likely than 66-75 year olds to rate their care very good or excellent, with the youngest patients (16-24 year olds) least likely to report excellent or very good overall care. Patients with any long-standing condition were less positive about their overall care; those with a learning disability or mental health condition were the least satisfied.

Clinical and trust-level characteristics were also associated with overall rating of care. With the exception of breast and skin cancer patients, all other patients were less likely than those with haematological cancers to rate their care as very good or excellent. Inpatients, patients who began their treatment more than one year ago and those who attended large acute trusts, trusts without foundation status or trusts with a "weak" CQC rating were also less likely to rate their care as very good or excellent.

Variation in patients' rating of care adjusting for clinical and trust-level factors

Model 1, shown in Table 3, shows the effect of mutually adjusting for all patient-level factors. The observed variation in rating under univariate logistic regression was mostly unaffected; negative associations between rating overall care positively and being female, younger, non-white or having a long-standing condition persisted. The magnitude of the associations was generally stable though there was a slight attenuation in the effect of having a mental health condition or learning disability and being of mixed ethnicity was no longer significantly associated. The addition of clinical factors (tumour group, time since first treatment and in- or day-patient status) to the regression model (Model 2) had little impact on variation by age or ethnicity, but the negative association between being female and care rating increased in magnitude. Including trust-level characteristics in the full multivariate model (Model 3) had a

For peer review only - http://bmjopen.bmj.com/site/about/guidelines.xhtml

minimal effect on the associations between patients' socio-demographic characteristics and rating of care. Even when adjusting for clinical, trust and other patient-level factors clear variation in patients' rating of care by socio-demographic characteristics such as gender, age, ethnicity and long-standing conditions was evident. Female, younger, non-White patients or patients with a long-standing condition remained less likely to rate their overall care as excellent or very good.

For peer review only

For peer review only - http://bmjopen.bmj.com/site/about/guidelines.xhtml

Table 3: Association between positive rating of overall care and demographic characteristics adjusting for patient, clinical and trust-level factors

	Univariate (from Table 2)		Multivariate Model 1[a]		Multivariate Model 2[b]		Multivariate Model 3[c]	
	OR (95% CI)	*p*-value	OR_{adj} (95% CI)	*p*-value	OR_{adj} (95% CI)	*p*-value	OR_{adj} (95% CI)	*p*-value
**Gender**								
Male	1 (ref)		1 (ref)		1 (ref)		1 (ref)	
Female	0.92 (0.88-0.96)	<0.001*	0.94 (0.89-0.99)	0.02*	0.72 (0.68-0.77)	<0.001*	0.72 (0.68-0.77)	<0.001*
**Age group**								
16 - 25	0.68 (0.51-0.92)	0.01*	0.69 (0.49-0.98)	0.04*	0.61 (0.43-0.88)	0.01*	0.58 (0.41-0.82)	0.002*
26 - 35	0.62 (0.52-0.75)	<0.001*	0.71 (0.60-0.86)	0.001*	0.64 (0.53-0.77)	<0.001*	0.62 (0.51-0.75)	<0.001*
36 - 50	0.70 (0.64-0.76)	<0.001*	0.72 (0.66-0.79)	<0.001*	0.61 (0.56-0.67)	<0.001*	0.60 (0.54-0.65)	<0.001*
51 - 65	0.79 (0.74-0.83)	<0.001*	0.79 (0.74-0.84)	<0.001*	0.74 (0.69-0.78)	<0.001*	0.72 (0.68-0.77)	<0.001*
66 - 75	1 (ref)		1 (ref)		1 (ref)		1 (ref)	
76+	0.85 (0.79-0.90)	<0.001*	0.86 (0.80-0.93)	<0.001*	0.91 (0.84-0.98)	0.02*	0.90 (0.84-0.98)	0.01*
**Ethnicity**								
White	1 (ref)		1 (ref)		1 (ref)		1 (ref)	
Mixed	0.66 (0.45-0.97)	0.04*	0.71 (0.47-1.07)	0.10	0.66 (0.43-1.01)	0.05	0.67 (0.43-1.04)	0.08
Asian	0.33 (0.29-0.38)	<0.001*	0.35 (0.30-0.41)	<0.001*	0.34 (0.29-0.41)	<0.001*	0.35 (0.30-0.41)	<0.001*
Black	0.41 (0.35-0.49)	<0.001*	0.43 (0.36-0.51)	<0.001*	0.43 (0.35-0.52)	<0.001*	0.43 (0.36-0.53)	<0.001*
Chinese	0.27 (0.19-0.39)	<0.001*	0.29 (0.20-0.42)	<0.001*	0.29 (0.20-0.43)	<0.001*	0.27 (0.18-0.41)	<0.001*
Other	0.58 (0.46-0.73)	<0.001*	0.59 (0.46-0.75)	<0.001*	0.59 (0.47-0.75)	<0.001*	0.61 (0.47-0.77)	<0.001*
**Long-standing conditions [d]**								
Deafness/hearing impairment	0.91 (0.85-0.98)	0.01*	0.92 (0.84-0.99)	0.04*	0.93 (0.85-1.01)	0.08	0.93 (0.85-1.01)	0.09
Blindness/visual impairment	0.74 (0.65-0.84)	<0.001*	0.81 (0.71-0.92)	0.001*	0.81 (0.71-0.92)	0.001*	0.86 (0.75-0.99)	0.03*
Physical condition	0.71 (0.67-0.76)	<0.001*	0.73 (0.69-0.77)	<0.001*	0.73 (0.69-0.77)	<0.001*	0.74 (0.70-0.78)	<0.001*
Learning disability	0.50 (0.39-0.65)	<0.001*	0.69 (0.52-0.91)	0.01*	0.71 (0.53-0.95)	0.02*	0.67 (0.50-0.90)	0.007*
Mental health condition	0.55 (0.48-0.64)	<0.001*	0.64 (0.56-0.72)	<0.001*	0.64 (0.57-0.74)	<0.001*	0.65 (0.57-0.74)	<0.001*
Long-standing illness	0.77 (0.73-0.82)	<0.001*	0.78 (0.73-0.84)	<0.001*	0.80 (0.75-0.86)	<0.001*	0.81 (0.75-0.87)	<0.001*

Calculations based on 60,528 respondents from 150 trusts with complete data
Abbreviations: OR=odds ratio; CI=confidence interval, ref=reference category

*Significant at α=0.05 level with clustered robust standard errors
[a] Adjusted for patient factors (i.e. ethnicity, gender, age group, specific long-standing conditions)
[b] Adjusted for patient factors and clinical factors (i.e. patient status, tumour group and time since first treatment)
[c] Adjusted for patient factors, clinical factors and trust-level factors (i.e. trust type, foundation status, CQC trust quality rating and quintile of frontline staff satisfied with care)
[d] Reference category for specific long-standing conditions is not having that condition

For peer review only - http://bmjopen.bmj.com/site/about/guidelines.xhtml

DISCUSSION

Our analysis of the 2011-2012 NCPES demonstrates that there is marked variation in the experiences of cancer patients by socio-demographic factors. Women, younger patients, ethnic minorities and patients with a long-standing condition or disability were less likely to rate their cancer care as "*excellent*" or "*very good*". This variation remained after adjusting for clinical factors, such as tumour group and duration of treatment, and trust-level factors. This suggests that the variation by socio-demographic factors is not a result of confounding but is attributable to real differences in experiences among these groups.

To the authors' knowledge, this was the first study to explore cancer patients' overall rating of care by socio-demographic characteristics including longstanding-conditions or disabilities. A principal strength of this study is the large sample size (> 71,000) and the response rate of 68%, which was significantly higher than that achieved by comparable surveys (22,23). Also, as all cancer patients treated by the NHS in England during the assigned 3 month study period were sent the survey, it is likely that findings can be generalised to the wider population of cancer patients. The main limitations of this study relate to the type of data available. The data for the trust quality score was collected approximately three years prior to the NCPES survey period and so may not reflect the quality of the trust at the time of patient admission. The influence of other potentially important predictors of patient experience such as employment status (20), level of deprivation (5) and health status (2,8,24) could not be explored as these data are not gathered through the NCPES. Furthermore, the binary categorisation of patients' responses, as per DH Survey Guidance, condenses the patients' experiences (which were already limited to several multiple choice options) and may mask potentially significant variation.

For peer review only - http://bmjopen.bmj.com/site/about/guidelines.xhtml

Interpretation of the findings from NCPES data requires consideration of the possible determinants of variation in patients' responses to a survey question. Firstly, it is possible that variation reflects differing health, emotional or other support needs that are not met by cancer care services (12,14,25). Secondly, differential expectations between patient groups, perhaps pertaining to socio-cultural norms, may contribute to the observed patterns (8,26). Thirdly, certain patient groups may have a tendency to respond less positively, based on shared norms regarding feedback and ideas as to its purpose (26). Finally, variation may reflect real differences in the quality of care provided (8,26).

Studies of patient experience in the general patient population have demonstrated systematic differences in experience by gender, age and ethnicity and the results of our study further add to this knowledge by demonstrating that similar variation exists among cancer patients. Adjusting for other socio-demographic factors, women were less likely to report positive experiences than men. This may be due to the increased emotional and support needs among female cancer patients described elsewhere (13,14). Breast cancer was the most common tumour group for females (38.4%, n=14,591) and in comparison to other tumour groups breast cancer patients were more likely to rate their care positively. Notably, when clinical factors such as tumour group were adjusted for, the magnitude of the negative association between gender and overall care rating increased. This may indicate that while patient experience varies modestly overall by gender there are marked differences between men and women with less common cancers. This is an area which merits further exploration.

For peer review only - http://bmjopen.bmj.com/site/about/guidelines.xhtml

Younger patients were less likely to report positive experiences than older patients, which corroborates previous findings in relation to age and patient satisfaction (4–8,13, 27). It has been suggested that this observation may reflect a generational phenomenon, whereby older patients' responses are influenced by comparisons with their parents' generation who may not have had access to advanced technologies of modern treatment or the free care provided by the NHS, referred to as 'gratitude bias' (28,29). Alternatively, younger patients may have higher expectations of quality of care due to a reduced frequency of hospital visits compared to older patients (17). The poorer rating of care in the oldest age group (76+ years) fits with neither theory and further work to understand the cause of the variation in cancer patient experience by age is required.

Ethnic minorities, especially Asian and specifically Chinese patients, reported less positive experiences than white patients. This trend is similar to findings from previous studies exploring variation in patient experiences of care generally (2,4,5) and specifically for cancer (17,22). The extent to which these results are due to cultural differences in expectations of care or willingness to criticise is unclear and necessitates further research. Of significant concern is the possibility that these patients experience poorer quality of care owing to a lack of understanding of the care needs of these minority groups or to discrimination, unintended or otherwise (8).

Patients with various long-standing conditions reported significantly less positive patient experiences than those without. The worst experiences were reported by patients with a learning disability or mental health condition. Given the small numbers of patients in these groups and the strength of the association it seems likely that there is marked variation in their experiences compared to other patients. Patients with long-

For peer review only - http://bmjopen.bmj.com/site/about/guidelines.xhtml

standing illnesses such as diabetes and chronic heart disease were also less likely to rate their care as “excellent” or “very good.” Given that the number of patients with such illnesses is set to rise in the future with an ageing patient population it is important to explore how having co-morbidities influences patients’ experience of cancer care.

This study presents evidence of inequalities in experiences of cancer care by gender, age, ethnicity and disability. Whilst it is possible that some of the variation observed between patient groups is a result of varying socio-cultural expectations or tendencies to rate care positively, it is also possible that the quality of care truly differs between patient groups. Further investigation of the experiences of women, ethnic minorities, younger patients and those with a disability is needed so that cancer care services can be better tailored to meet the needs and expectations of these groups. Analysis of the NCPES qualitative free text questions and other patient experience data at a trust level would help to inform quality improvement initiatives. The findings of this study would appear to suggest that, if used as a comparative performance indicator (as is NCPES data) patient experience measures should be adjusted for age, gender and ethnicity. An unadjusted measure of performance could unfairly disadvantage hospital trusts with higher than average proportion of ethnic minority patients, for example. However, the impact of adjusting NCPES data for demographic characteristics on trust rankings has been shown to be minimal (30). Adjusting for gender, age and ethnicity causes few trusts to move into or out of the top or bottom 20% of trusts nationally. While they may not account for much of the between-trust variation in cancer patient experience the overall variation in patient experience by demographic factors is important in its own right and warrants further attention.

For peer review only

For peer review only - http://bmjopen.bmj.com/site/about/guidelines.xhtml

Responses to survey questions are a result of patients' perception and interpretation of events, which are shaped by expectations and clinical or emotional needs, in addition to the quality of services received. Meeting the care needs of all patients equally is a fundamental principle of the NHS and high-quality cancer services must strive to meet the needs of its diverse patient population. This study identifies patient groups for which cancer care services are in greatest need of improvement.

KEY MESSAGES (3-4 sentences less than 25 words each)

- Adjusting for clinical and trust-level factors, there is evidence of inequalities in patients' experiences of cancer care by socio-demographic characteristics.
- Female, younger and non-white (especially Asian) cancer patients are less likely to rate their overall care as excellent or very good.
- Patients with a long-standing condition also report poorer overall care, patients with a learning disability or mental health condition being least satisfied.
- While patient experience varies modestly overall by gender, there may be marked differences between the experiences of men and women with less common cancers.

For peer review only - http://bmjopen.bmj.com/site/about/guidelines.xhtml

LICENCE FOR PUBLICATION STATEMENT

The Corresponding Author has the right to grant on behalf of all authors and does grant on behalf of all authors, an exclusive licence (or non-exclusive for government employees) on a worldwide basis to the BMJ Group and co-owners or contracting owning societies (where published by the BMJ Group on their behalf), and its Licensees to permit this article (if accepted) to be published in Sexually Transmitted Infections and any other BMJ Group products and to exploit all subsidiary rights, as set out in our licence http://group.bmj.com/products/journals/instructions-for-authors/licence-forms.

For peer review only

Acknowledgements: None

Contributorship statement: HW devised the study, advised on data analysis and participated in interpreting the data and reviewing the manuscript; SD participated in interpreting the data and reviewing the manuscript; LM carried out supplementary data analysis, participated in interpreting the data and co-prepared the manuscript; AB carried out the statistical analysis, interpreted the data and co-prepared the manuscript.

Funding: There was no specific funding for this project. HW, SD and LM are supported by the NIHR Imperial College Healthcare NHS Trust Biomedical Research Centre and Imperial College Healthcare Charity.

Competing interests: None

Ethics: No ethical approval was required for this study

Data sharing: The National Cancer Patient Experience Survey 2012-13 dataset used is available for download from http://ukdataservice.ac.uk.

For peer review only - http://bmjopen.bmj.com/site/about/guidelines.xhtml

REFERENCES

1. NHS England. Putting patients first: The NHS England business plan for 2013/14 – 2015/16. 2012 p. 1–64.

2. Healthcare Commission. Variations in the experiences of patients in England: Analysis of the Healthcare Commission's 2003/2004 national surveys of patients. London; 2005 p. 1–35.

3. Department of Health. Report on the self reported experience of patients from black and minority ethnic groups. London; 2009 p. 1–31.

4. Campbell JL, Ramsay J, Green J. Age, gender, socioeconomic, and ethnic differences in patients' assessments of primary health care. *Qual Health Care* 2001;**10**(2):90–5.

5. Commission for Health Improvement. Unpacking the patients' perspective: Variations in NHS patient experience in England. London; 2004 p. 1–112.

6. Sizmur S. Multilevel analysis of inpatient experience. Picker Institute Europe. 2011 p. 1–28.

For peer review only - http://bmjopen.bmj.com/site/about/guidelines.xhtml

7. Jenkinson C, Coulter A, Bruster S, et al. Patients' experiences and satisfaction with health care: results of a questionnaire study of specific aspects of care. *Qual Saf Health Care* 2002;**11**(4):335–9.

8. Lyratzopoulos G, Elliott M, Barbiere JM, et al. Understanding ethnic and other socio-demographic differences in patient experience of primary care: evidence from the English General Practice Patient Survey. *BMJ Qual Saf* 2012;**21**(1):21–9.

9. Mead N, Roland M. Understanding why some ethnic minority patients evaluate medical care more negatively than white patients: a cross sectional analysis of a routine patient survey in English general practices. *BMJ* 2009;**339**:b3450.

10. Department of Health. Improving Outcomes: A Strategy for Cancer. 2011 p. 1–99.

11. Davidson R, Mills ME. Cancer patients' satisfaction with communication, information and quality of care in a UK region. *Eur J Cancer Care (Engl)* 2005;**14**(1):83–90.

12. Cox A, Jenkins V, Catt S, et al. Information needs and experiences: an audit of UK cancer patients. *Eur J Oncol Nurs* 2006;**10**(4):263–72.

13. Clarke S, Booth L, Velikova G, et al. Social support: gender differences in cancer patients in the United Kingdom. *Cancer Nurs* 2006;**29**(1):66–72.

14. Morrison V, Henderson BJ, Zinovieff F, et al. Common, important, and unmet needs of cancer outpatients. *Eur J Oncol Nurs* 2012;**16**:115–23.

For peer review only - http://bmjopen.bmj.com/site/about/guidelines.xhtml

15. Department of Health. Cancer Patient Experience Survey 2011/12: National Report. 2011 p. 1–168.

16. Saunders CL, Abel GA, El Turabi A, et al. Accuracy of routinely recorded ethnic group information compared with self-reported ethnicity: evidence from the English Cancer Patient Experience Survey. *BMJ Open*. 2013;**3**(6):1–9.

17. El Turabi A, Abel GA, Roland M, et al. Variation in reported experience of involvement in cancer treatment decision making: evidence from the National Cancer Patient Experience Survey. *Br J Cancer* 2013;**109**(3):780–7.

18. Ipsos MORI. Frontiers of performance in the NHS II. 2008 p. 1–100.

19. Raleigh VS, Frosini F, Sizmur S, et al. Do some trusts deliver a consistently better experience for patients? An analysis of patient experience across acute care surveys in English NHS trusts. *BMJ Qual Saf* 2012;**21**(5):381–90.

20. Kontopantelis E, Roland M, Reeves D. Patient experience of access to primary care: identification of predictors in a national patient survey. BMC Fam Pract 2010;**11**(61):1–15.

21. Kreft IG, de Leeuw J. Introducing Multilevel Modeling. London: SAGE Publications; 2002.

22. Lyratzopoulos G, Neal RD, Barbiere JM, et al. Variation in number of general practitioner consultations before hospital referral for cancer: findings from the 2010 National Cancer Patient Experience Survey in England. *Lancet Oncol* 2012;**13**(4):353–65.

For peer review only

23. Care Quality Commission. National NHS patient survey programme: Survey of adult inpatients 2012. 2013 p. 1–34.

24. Cohen G. Age and health status in a patient satisfaction survey. *Soc Sci Med* 1996;**42**(7):1085–93.

25. Pinquart M, Duberstein PR. Information needs and decision-making processes in older cancer patients. *Crit Rev Oncol Hematol* 2004;**51**(1):69–80.

26. Perneger T. Adjustment for patient characteristics in satisfaction surveys. *Int J Qual Heal Care* 2004;**16**(6):433–5.

27. Crow R, Gage H, Hampson S, et al. The measurement of satisfaction practice from a systematic review. *Health Technol Assess* 2002;**6**(32):1–250.

28. Department of Health. Methods, reasoning and scope: Statement of methodology for the overall patient experience scores. 2013 p. 1–28.

29. Bowling A. An "inverse satisfaction law"? Why don't older patients criticise health services? *J Epidemiol Community Health* 2002;**56**(7):482.

30. Saunders K, Lyratzopoulos G, Abel G. Is case-mix adjustment important for the National Cancer Patient Experience Survey in England? Cambridge Centre for Health Services Research; 2013 p. 1–12.

For peer review only

For peer review only - http://bmjopen.bmj.com/site/about/guidelines.xhtml

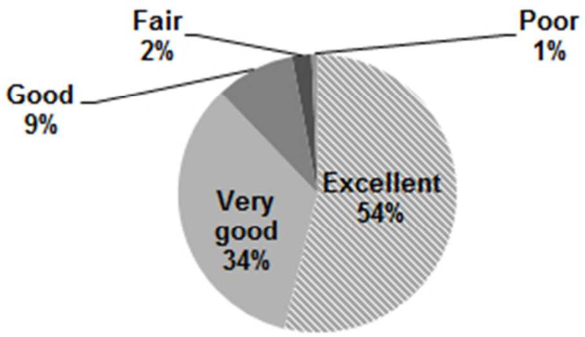

For peer review only

STROBE Statement—Checklist of items that should be included in reports of ***cross-sectional studies***

	Item No	Recommendation		Note
**Title and abstract**	1	(*a*) Indicate the study's design with a commonly used term in the title or the abstract	✓	
		(*b*) Provide in the abstract an informative and balanced summary of what was done and what was found	✓	
**Introduction**				
Background/rationale	2	Explain the scientific background and rationale for the investigation being reported	✓	
Objectives	3	State specific objectives, including any pre-specified hypotheses	✓	
**Methods**				
Study design	4	Present key elements of study design early in the paper	✓	
Setting	5	Describe the setting, locations, and relevant dates, including periods of recruitment, exposure, follow-up, and data collection	✓	
Participants	6	(*a*) Give the eligibility criteria, and the sources and methods of selection of participants	✓	
Variables	7	Clearly define all outcomes, exposures, predictors, potential confounders, and effect modifiers. Give diagnostic criteria, if applicable	✓	
Data sources/ measurement	8*	For each variable of interest, give sources of data and details of methods of assessment (measurement). Describe comparability of assessment methods if there is more than one group	✓	
Bias	9	Describe any efforts to address potential sources of bias	N/A	No weights available
Study size	10	Explain how the study size was arrived at	N/A	Response rate described
Quantitative variables	11	Explain how quantitative variables were handled in the analyses.<br>If applicable, describe which groupings were chosen and why	✓<br>✓	
Statistical methods	12	(*a*) Describe all statistical methods, including those used to control for confounding	✓	
		(*b*) Describe any methods used to examine subgroups and interactions	N/A	No sub-analyses done
		(*c*) Explain how missing data were addressed	✓	
		(*d*) If applicable, describe analytical methods taking account of sampling strategy	✓	
		(*e*) Describe any sensitivity analyses	N/A	No sensitivity analyses done
**Results**				
Participants	13*	(a) Report numbers of individuals at each stage of study—eg numbers potentially eligible, examined for eligibility, confirmed eligible, included in the study, completing follow-up, and analysed	✓	

For peer review only - http://bmjopen.bmj.com/site/about/guidelines.xhtml

		(b) Give reasons for non-participation at each stage	N/A	
		(c) Consider use of a flow diagram	N/A	
Descriptive data	14*	(a) Give characteristics of study participants (eg demographic, clinical, social) and information on exposures and potential confounders	✓	
		(b) Indicate number of participants with missing data for each variable of interest	✓	
Outcome data	15*	Report numbers of outcome events or summary measures	✓	
Main results	16	(*a*) Give unadjusted estimates and, if applicable, confounder-adjusted estimates and their precision (eg, 95% confidence interval). Make clear which confounders were adjusted for and why they were included	✓	
		(*b*) Report category boundaries when continuous variables were categorized	✓	
		(*c*) If relevant, consider translating estimates of relative risk into absolute risk for a meaningful time period	N/A	
Other analyses	17	Report other analyses done—eg analyses of subgroups and interactions, and sensitivity analyses	N/A	
**Discussion**				
Key results	18	Summarise key results with reference to study objectives	✓	
Limitations	19	Discuss limitations of the study, taking into account sources of potential bias or imprecision. Discuss both direction and magnitude of any potential bias	✓	
Interpretation	20	Give a cautious overall interpretation of results considering objectives, limitations, multiplicity of analyses, results from similar studies, and other relevant evidence	✓	
Generalisability	21	Discuss the generalisability (external validity) of the study results	✓	
**Other information**				
Funding	22	Give the source of funding and the role of the funders for the present study and, if applicable, for the original study on which the present article is based	✓	

*Give information separately for exposed and unexposed groups.

Note: An Explanation and Elaboration article discusses each checklist item and gives methodological background and published examples of transparent reporting. The STROBE checklist is best used in conjunction with this article (freely available on the Web sites of PLoS Medicine at http://www.plosmedicine.org/, Annals of Internal Medicine at http://www.annals.org/, and Epidemiology at http://www.epidem.com/). Information on the STROBE Initiative is available at www.strobe-statement.org.

For peer review only - http://bmjopen.bmj.com/site/about/guidelines.xhtml

Inequalities in cancer patients' experiences of care: analysis of data from the National Cancer Patient Experience Survey 2011-12

Journal:	*BMJ Open*
Manuscript ID:	bmjopen-2013-004567.R1
Article Type:	Research
Date Submitted by the Author:	09-Jan-2014
Complete List of Authors:	Bone, Anna; Imperial College London, School of Public Health<br>Mc Grath-Lone, Louise; Imperial College London, Patient Experience Research Centre, School of Public Health<br>Day, Sophie; Imperial College London, Patient Experience Research Centre, School of Public Health<br>Ward, Helen; Imperial College London, Patient Experience Research Centre, School of Public Health
**Primary Subject Heading**:	Public health
Secondary Subject Heading:	Oncology
Keywords:	PUBLIC HEALTH, ONCOLOGY, Adult oncology < ONCOLOGY

SCHOLARONE™ Manuscripts

For peer review only - http://bmjopen.bmj.com/site/about/guidelines.xhtml

Inequalities in cancer patients' experiences of care: analysis of data from the National Cancer Patient Experience Survey 2011-12

Corresponding author: Louise Mc Grath-Lone, Patient Experience Research Centre, School of Public Health, Imperial College London, W2 1PG, UK.

E-mail:Louise.mc-grath-lone@imperial.ac.uk

Phone: 0207 5943417 Fax: 0207 4023927

Authors: Anna Bone[1], Louise Mc Grath-Lone[2], Sophie Day[2], Helen Ward.[2]

Affiliations:

1. School of Public Health, Imperial College London, W2 1PG, UK.
2. Patient Experience Research Centre, School of Public Health, Imperial College, London, W2 1PG, UK.

Keywords: Patient satisfaction, quality measurement, surveys

Word count: 2,912

For peer review only

ABSTRACT

Background

Patient experience is a key component of quality in healthcare; however, studies in the UK indicate that patients' experience of care is not equal across gender, age and ethnicity. There are currently 1.8 million people living with cancer in the UK but less is known about their experiences of care. Whether inequalities by socio-demographic characteristics exist amongst cancer patients is not well-documented.

Methods

Using cross-sectional data from 71,793 cancer patients who completed the National Cancer Patient Experience Survey 2011-2012, we examined associations between patient, clinical and trust-level factors and a summary measure of patient experience, namely overall rating of care. Multivariate logistic regression was used to investigate variation by socio-demographic characteristics adjusting for other patient, clinical and trust-level factors.

Results

Female, non-white and younger patients were less likely to rate their overall care as excellent or very good. Patients with long-standing conditions, particularly those with learning disabilities or mental health conditions also reported poorer overall care. This variation persisted when other patient, clinical and trust-level factors were controlled

For peer review only

for, indicating there are real differences in experiences among cancer patients by socio-demographic characteristics.

Conclusion

There is evidence of inequalities in cancer patient experience in the UK by socio-demographic characteristics such as gender, age, ethnicity and disability. Quality cancer care services must strive to meet the needs of a diverse patient population equally; this study identifies patient groups for whom it appears cancer care services are in greatest need of improvement.

Article summary: Strengths and limitations of this study

- To the authors' knowledge, this is the first study to explore cancer patients' overall rating of care by socio-demographic characteristics including longstanding-conditions or disabilities.
- A principal strength of this study is the large sample size (> 71,000) and high response rate (68%).
- As this study involves secondary analysis of national survey data it is limited by the type of data available e.g. the influence of potentially important predictors of patient experience such as employment status, level of deprivation and health status could not be explored as these data were not gathered.
- A further limitation is that the binary categorisation of patients' responses condenses the patients' experiences (which were already limited to several multiple choice options) and may mask potentially significant variation.

For peer review only - http://bmjopen.bmj.com/site/about/guidelines.xhtml

For peer review only

INTRODUCTION

Patient experience is a key component of quality in healthcare and is one of the top priorities in the NHS (1). However, studies in the UK indicate that there are systematic differences in patient experience by socio-demographic characteristics (2–4). For example, studies on primary and hospital care have found that patients tend to report more positive experiences with increasing age (4–8), females report less positive experiences than males (5,6) and non-white patients report less positive experiences than white patients, even after adjusting for other socio-demographic variables (3,5,6,9). Less is known about variation in the experiences of cancer patients. There are currently 1.8 million people living with cancer in the UK (10) and advances in cancer treatments mean that they are living longer and facing prolonged periods of contact with healthcare services because of complex treatment regimens (11). In 2011, a Department of Health (DH) report set out the government's strategy to improve outcomes by putting patients at the heart of cancer health services (10). A key objective of this strategy was to reduce inequalities in care relating to both clinical outcomes and patient experience. Thus, exploration of the experiences of cancer patients and the inequalities that may exist is critical in order to identify patient groups for whom cancer care services are in greatest need of improvement.

Detailed studies on the experiences of cancer patients have often relied on small sample sizes (12–14). However, a regular National Cancer Patient Experience Survey (NCPES) has been established which provides a wealth of information on care and treatment experiences. The 2011-12 survey includes responses from over 71,000 cancer patients from 160 trusts across the UK (15). With a relatively high response rate and large sample size, the survey presents an opportunity to explore inequalities

For peer review only - http://bmjopen.bmj.com/site/about/guidelines.xhtml

in the quality of care received by cancer patients. Here, we aim to describe the variation in cancer patient experience by age, gender, ethnicity and presence of long-standing conditions or disabilities in order to explore whether there are systematic inequalities. We further examine the influence of clinical and trust-level factors on these variations.

METHODS

Source of data

We carried out a secondary analysis of 2011-2012 NCPES cross-sectional data collected by Quality Health (Chesterfield, UK) on behalf of the DH. All patients with a primary diagnosis of cancer who attended an NHS hospital as an inpatient or day case between 1st September 2011 and 30th November 2011 were sent the survey (15). Non-responders were followed up with two postal reminders. The final response rate achieved was 68%. As no survey weights were available, the data could not be weighted to adjust for non-response. The dataset included demographic and clinical characteristics for 71,793 cancer patients who attended 160 hospital trusts across England, as well as their responses to 70 multiple choice questions relating to various aspects of their experiences of care. Surveys such as NCPES are commonly used to measure patient experience over a range of domains; however, single summary measures of overall experience, such as the Family and Friends Test, have become increasingly important (1). Our analysis focused on a summary measure of patient experience, namely patients' assessment of care as measured by Q70 in the survey, *"Overall, how would you rate your care?"* Responses from a five point scale were transformed into a binary outcome, with "*excellent"* and "*very good"* categorised as

For peer review only - http://bmjopen.bmj.com/site/about/guidelines.xhtml

'positive' and *"good"*, *"fair"* and *"poor"* as 'not positive', in accordance with the DH Survey Guidance (15).

Patient, clinical and trust-level characteristics

The main socio-demographic characteristics of interest in this study (gender, age and ethnicity) were ascertained by self-report (16) and grouped as in the national report (15). As Chinese patients have reported less positive experiences than white patients elsewhere (3,6,17), the "Chinese" ethnic category was not combined with "Other" in this analysis. For age and ethnicity the largest groups were chosen as the reference category. Responses to the question "*Do you have any of the following long-standing conditions?"* were used to identify patients with co-morbidities. The clinical characteristics of tumour group and patient status (i.e. day or inpatient) were taken from hospital administration records.

Haematological cancer patients were assigned as the reference tumour group as the largest group (breast) did not have a representative age and gender distribution. Time since first treatment was ascertained by patients' survey responses. As trust-level factors have previously been associated with patient experience (6,18–20) several were included in this analysis. Hospital trusts were categorised by type (large acute, medium acute, small acute, specialist and teaching) and by foundation status. The Care Quality Commission's (CQCs) 2008/2009 Annual Health Check rating and the proportion of frontline staff satisfied with care at their trust (Q12d from the National NHS Staff Survey 2012) were also included as measures of trusts' overall quality. Quintiles of staff satisfaction were used as a categorical variable during regression

For peer review only - http://bmjopen.bmj.com/site/about/guidelines.xhtml

analysis with the lowest quintile as reference category. The reference categories chosen for other trust-level factors were the largest groups.

Data analysis

Variation in patents' overall rating of care by patient, clinical and trust-level factors was investigated using univariate logistic regression. Respondents with missing demographic, clinical or trust-level data, or those who did not answer Q70, were then excluded (i.e. complete-case analysis was undertaken) and multivariate logistic regression was used to describe associations between the individual demographic characteristics of interest and overall rating of care. Confounding by patient, clinical and trust-level factors was controlled for through their sequential addition to the model. Logistic regression was chosen as the small intra-class coefficient calculated for Q70 (<0.01) suggested the effect of clustering by trust among respondents was negligible; therefore, it was anticipated that a multilevel model and a multivariate logistic regression model would produce similar results (21). However, as even small intra-class correlations can inflate type-1 errors, clustered robust standard errors were utilised. All statistical analyses were conducted in Stata V.12.

For peer review only - http://bmjopen.bmj.com/site/about/guidelines.xhtml

RESULTS

Patient characteristics and rating of care

A total of 71,793 patients admitted to hospital trusts across England with a primary cancer diagnosis completed the survey. Table 1 shows the demographic and clinical characteristics of respondents and Table 2 the characteristics of the hospital trusts they attended. The majority of patients were white, female and >50 years old and there were substantial numbers with disabilities or other long-standing conditions. The most common tumour groups were breast and haematological cancers. Most respondents had started their treatment in the last year and were admitted to hospital as day case patients on their most recent visit. Most were treated in large acute trusts, trusts with foundation status and trusts rated 'Good' by CQC. The majority of patients (96.5%, n=69,276) provided a response to Q70 "*Overall, how would you rate your care?*" In total 87.8% rated their care as "*excellent*" or "*very good*" while the remaining 12.2% rated their care "*good*", "*fair*" or "*poor*" (Fig.1).

For peer review only

Table 1: Characteristics of survey respondents and their unadjusted associations with a positive overall rating of care from univariate logistic regression

Patient characteristics					Clinical characteristics				
**Gender**	n	%	OR (95% CI)	*p*-value	**Tumour group**	n	%	OR (95% CI)	*p*-value
Male	33,832	47.1	1 (ref)		Brain/CNS	746	1.0	0.52 (0.42-0.63)	**<0.001**
Female	37,961	52.9	0.92 (0.88-0.96)	**<0.001**	Breast	14,739	20.5	1.00 (0.92-1.09)	0.98
					Colorectal / Lower GI	9,483	13.2	0.72 (0.66-0.78)	**<0.001**
**Age group**	n	%	OR (95% CI)	*p*-value	Gynaecological	4,202	5.9	0.72 (0.64-0.80)	**<0.001**
16 - 25	363	0.5	0.68 (0.51-0.92)	**0.01**	Haematological	11,070	15.4	1 (ref)	
26 - 35	969	1.4	0.62 (0.52-0.75)	**<0.001**	Head and Neck	2,422	3.4	0.71 (0.62-0.81)	**<0.001**
36 - 50	6,802	9.5	0.70 (0.64-0.76)	**<0.001**	Lung	5,029	7.0	0.77 (0.69-0.85)	**<0.001**
51 - 65	22,885	31.9	0.79 (0.74-0.83)	**<0.001**	Other	1,138	1.6	0.72 (0.59-0.86)	**<0.001**
66 - 75	23,643	32.9	1 (ref)		Prostate	5,831	8.1	0.70 (0.64-0.78)	**<0.001**
76+	17,131	23.9	0.85 (0.79-0.90)	**<0.001**	Sarcoma	2,451	3.4	0.61 (0.53-0.69)	**<0.001**
					Skin	1,695	2.4	0.98 (0.82-1.16)	0.80
**Ethnicity**	n	%	OR (95% CI)	*p*-value	Upper GI	4,540	6.3	0.61 (0.55-0.68)	**<0.001**
White	63,652	88.7	1 (ref)		Urological	8,447	11.8	0.64 (0.58-0.70)	**<0.001**
Mixed	199	0.3	0.66 (0.45-0.97)	**0.04**					
Asian/Asian British	1,082	1.5	0.33 (0.29-0.38)	**<0.001**	**Patient status**	n	%	OR (95% CI)	*p*-value
Black/Black British	885	1.2	0.41 (0.35-0.49)	**<0.001**	Day case	45,720	63.7	1 (ref)	
Chinese	138	0.2	0.27 (0.19-0.39)	**<0.001**	Inpatient	26,073	36.3	0.84 (0.80-0.88)	**<0.001**
Other	510	0.7	0.58 (0.46-0.73)	**<0.001**					
					**Time since first treatment**	n	%	OR (95% CI)	*p*-value
**Long-standing conditions** [a, b]	n	%	OR (95% CI)	*p*-value	< 1 year	44,997	62.3	1 (ref)	
None	48,218	67.2			1-5 years	17,486	24.4	0.83 (0.78-0.87)	**<0.001**
Deafness/hearing impairment	7,281	10.1	0.91 (0.85-0.98)	**0.01**	>5 years	6,212	8.7	0.88 (0.81-0.95)	**0.002**
Blindness/partially sighted	1,856	2.6	0.74 (0.65-0.84)	**<0.001**					
Physical condition	9,347	13.0	0.71 (0.67-0.76)	**<0.001**					
Learning disability	354	0.5	0.50 (0.39-0.65)	**<0.001**					
Mental health condition	1,347	1.9	0.55 (0.48-0.64)	**<0.001**					
Long-standing illness [c]	9,241	12.9	0.77 (0.73-0.82)	**<0.001**					

Total number of respondents=71,793. Ethnicity was unknown for 7.4% respondents, long-standing conditions status for 7.3% and time since first treatment for 4.3%. Significant associations at α=0.05 level highlighted in bold.

[a] 6.7% of patients (n=4,780) had >1 long-standing condition, therefore the column total exceeds 100%
[b] Reference category for specific long-standing conditions is not having that condition
[c] Such as (but not limited to) HIV, diabetes, chronic heart disease or epilepsy

For peer review only

For peer review only - http://bmjopen.bmj.com/site/about/guidelines.xhtml

Table 2: Characteristics of trusts attended by survey respondents and their unadjusted associations with a positive overall rating of care from univariate logistic regression

Trust-level characteristics				
**Trust type**	n	%	OR (95% CI)	*p*-value
Small acute	6,240	8.7	1.23 (1.12-1.34)	**<0.001**
Medium acute	16,677	23.2	1.07 (1.01-1.14)	**0.02**
Large acute	25,850	36.0	1 (ref)	
Specialist	3,224	4.5	1.54 (1.36-1.76)	**<0.001**
Teaching	19,802	27.6	1.03 (0.98-1.10)	0.24
**Foundation status**	n	%	OR (95% CI)	*p*-value
No	31,798	44.3	0.82 (0.78-0.85)	**<0.001**
Yes	39,995	55.7	1 (ref)	
**CQC trust quality rating (2008/9)**	n	%	OR (95% CI)	*p*-value
Weak	3,926	5.6	0.85 (0.77-0.94)	**0.001**
Fair	18,482	26.2	0.97 (0.92-1.03)	0.34
Good	28,425	40.3	1 ref	
Excellent	19,748	28.0	1.17 (1.10-1.24)	**<0.001**

**Frontline staff satisfied with care** [a]		**Quintiles of frontline staff satisfied with care** [b]		
			OR (95% CI)	*p*-value
Mean	63.5%			
Median	62.7%	1 (lowest)	1 (ref)	
Range	35.3 - 94.0%	2	1.10 (1.03-1.19)	**0.01**
		3	1.11 (1.03-1.19)	**0.004**
		4	1.17 (1.09-1.26)	**<0.001**
		5 (highest)	1.35 (1.25-1.45)	**<0.001**

Total number of respondents=71,793. CQC trust quality rating was unknown for 3 trusts (1.7% of respondents)and the proportion of frontline staff satisfied with care was unknown for 1 trust (1.7% of respondents).Significant associations at α=0.05 level highlighted in bold.

[a] Calculated from responses to Q12d from the National NHS Staff Survey 2012
[b] Trusts were categorised into quintiles according to the proportion of staff responding positively to Q12d

Abbreviations: CQC=Care Quality Commission

Variation in rating of care by patient, clinical and trust-level factors

Unadjusted associations between patient, clinical and trust-level characteristics and Q70 from univariate logistic regression analysis are shown in Tables 1 & 2. Among all respondents, statistically significant variation in overall rating of care by patient-level characteristics such as ethnicity, gender, age and long-standing conditions was observed. For example, women were less likely than men, and non-white patients were less likely than white patients, to rate their overall care very good or excellent. Chinese patients reported least favourably among non-white ethnic minorities.

For peer review only - http://bmjopen.bmj.com/site/about/guidelines.xhtml

For peer review only

Younger and older patients were less likely than 66-75 year olds to rate their care very good or excellent, with the youngest patients (16-24 year olds) least likely to report excellent or very good overall care. Patients with any long-standing condition were less positive about their overall care; those with a learning disability or mental health condition were the least satisfied.

Clinical and trust-level characteristics were also associated with overall rating of care. With the exception of breast and skin cancer patients, all other patients were less likely than those with haematological cancers to rate their care as very good or excellent. Inpatients, patients who began their treatment more than one year ago and those who attended large acute trusts, trusts without foundation status or trusts with a "weak" CQC rating were also less likely to rate their care as very good or excellent.

Variation in patients' rating of care adjusting for clinical and trust-level factors

After excluding those with missing demographic, clinical or trust-level data, or those who did not provide a rating of their overall care, 60,528 respondents from 150 trusts remained for complete-case analysis. The distribution of patient, clinical and trust-level characteristics in the "complete-case" and "all respondents" populations was similar (Supplemetary Table 1) and there was little difference in the univariate associations between the demographic characteristics and overall rating of care (with the exception of being deaf/having a hearing impairment which was not associated with a poorer rating of overall care during complete-case analysis, Table 3). Model 1 in Table 3 shows the effect of mutually adjusting for all patient-level factors. The observed variation in rating under univariate logistic regression was mostly unaffected; negative

For peer review only - http://bmjopen.bmj.com/site/about/guidelines.xhtml

associations between rating overall care positively and being female, younger, non-white or having a long-standing condition persisted. The magnitude of the associations was generally stable though there was a slight increase in the effect of having a mental health condition or learning disability and being of mixed ethnicity was no longer significantly associated. The addition of clinical factors (tumour group, time since first treatment and in- or day-patient status) to the regression model (Model 2) had little impact on variation by age or ethnicity, but the negative association between being female and care rating increased in magnitude. Including trust-level characteristics in the full multivariate model (Model 3) had a minimal effect on the associations between patients' socio-demographic characteristics and rating of care. Even when adjusting for clinical, trust and other patient-level factors clear variation in patients' rating of care by socio-demographic characteristics such as gender, age, ethnicity and long-standing conditions was evident. Female, younger, non-White patients or patients with a long-standing condition remained less likely to rate their overall care as excellent or very good.

For peer review only

For peer review only - http://bmjopen.bmj.com/site/about/guidelines.xhtml

Table 3: Association between positive rating of overall care and demographic characteristics adjusting for patient, clinical and trust-level factors

	Univariate [a]		Multivariate Model 1 [b]		Model 2 [c]		Model 3 [d]	
	OR (95% CI)	*p*-value	OR_{adj} (95% CI)	*p*-value	OR_{adj} (95% CI)	*p*-value	OR_{adj} (95% CI)	*p*-value
**Gender**								
Male	1 (ref)		1 (ref)		1 (ref)		1 (ref)	
Female	0.91 (0.87-0.96)	**<0.001**	0.93 (0.88-0.98)	**0.02**	0.72 (0.68-0.76)	**<0.001**	0.72 (0.68-0.77)	**<0.001**
**Age group**								
16 - 25	0.65 (0.48-0.90)	**0.01**	0.68 (0.48-0.98)	**0.04**	0.61 (0.43-0.88)	**0.01**	0.58 (0.41-0.82)	**0.002**
26 - 35	0.67 (0.55-0.81)	**<0.001**	0.71 (0.60-0.86)	**0.001**	0.64 (0.53-0.77)	**<0.001**	0.62 (0.51-0.75)	**<0.001**
36 - 50	0.68 (0.63-0.75)	**<0.001**	0.71 (0.65-0.78)	**<0.001**	0.61 (0.56-0.67)	**<0.001**	0.60 (0.54-0.65)	**<0.001**
51 - 65	0.76 (0.72-0.81)	**<0.001**	0.77 (0.73-0.82)	**<0.001**	0.73 (0.68-0.77)	**<0.001**	0.72 (0.68-0.77)	**<0.001**
66 - 75	1 (ref)		1 (ref)		1 (ref)		1 (ref)	
76+	0.85 (0.79-0.91)	**<0.001**	0.86 (0.80-0.93)	**<0.001**	0.90 (0.83-0.97)	**0.01**	0.90 (0.84-0.98)	**0.01**
**Ethnicity**								
White	1 (ref)		1 (ref)		1 (ref)		1 (ref)	
Mixed	0.65 (0.44-0.97)	**0.04**	0.70 (0.45-1.10)	0.12	0.68 (0.44-1.08)	0.10	0.67 (0.43-1.04)	0.08
Asian	0.35 (0.30-0.40)	**<0.001**	0.37 (0.31-0.44)	**<0.001**	0.35 (0.30-0.42)	**<0.001**	0.35 (0.30-0.41)	**<0.001**
Black	0.45 (0.37-0.53)	**<0.001**	0.46 (0.38-0.56)	**<0.001**	0.46 (0.37-0.56)	**<0.001**	0.43 (0.36-0.53)	**<0.001**
Chinese	0.26 (0.18-0.38)	**<0.001**	0.28 (0.19-0.41)	**<0.001**	0.29 (0.20-0.42)	**<0.001**	0.27 (0.18-0.41)	**<0.001**
Other	0.61 (0.47-0.77)	**<0.001**	0.62 (0.48-0.79)	**<0.001**	0.61 (0.47-0.79)	**<0.001**	0.61 (0.47-0.77)	**<0.001**
**Long-standing conditions [e]**								
Deafness/hearing impairment	0.95 (0.87-1.02)	0.16	0.93 (0.85-0.99)	0.09	0.93 (0.85-1.01)	0.09	0.93 (0.85-1.01)	0.09
Blindness/visual impairment	0.78 (0.68-0.90)	**0.001**	0.85 (0.74-0.97)	**0.01**	0.85 (0.75-0.98)	**<0.001**	0.86 (0.75-0.99)	**0.03**
Physical condition	0.73 (0.68-0.78)	**<0.001**	0.73 (0.69-0.77)	**<0.001**	0.73 (0.69-0.77)	**<0.001**	0.74 (0.70-0.78)	**<0.001**
Learning disability	0.49 (0.38-0.65)	**<0.001**	0.66 (0.49-0.88)	**0.01**	0.68 (0.51-0.90)	**0.01**	0.67 (0.50-0.90)	**0.01**
Mental health condition	0.58 (0.50-0.67)	**<0.001**	0.65 (0.57-0.74)	**<0.001**	0.65 (0.57-0.74)	**<0.001**	0.65 (0.57-0.74)	**<0.001**
Long-standing illness	0.79 (0.74-0.85)	**<0.001**	0.79 (0.74-0.85)	**<0.001**	0.80 (0.75-0.86)	**<0.001**	0.81 (0.75-0.87)	**<0.001**

Significant associations at α=0.05 level with clustered robust standard errors highlighted in bold.

[a] Figures may differ from those presented in Tables 1 & 2 as they are based on 60,528 respondents from 150 trusts with complete data (i.e. complete-case analysis)
[b] Adjusted for patient factors (i.e. ethnicity, gender, age group, specific long-standing conditions)
[c] Adjusted for patient factors and clinical factors (i.e. patient status, tumour group and time since first treatment)
[d] Adjusted for patient factors, clinical factors and trust-level factors (i.e. trust type, foundation status, CQC trust quality rating and quintile of frontline staff satisfied with care)
[e] Reference category for specific long-standing conditions is not having that condition Abbreviations: OR=odds ratio; CI=confidence interval, ref=reference category

For peer review only - http://bmjopen.bmj.com/site/about/guidelines.xhtml

DISCUSSION

Our analysis of the 2011-2012 NCPES demonstrates that there is marked variation in the experiences of cancer patients by socio-demographic factors. Women, younger patients, ethnic minorities and patients with a long-standing condition or disability were less likely to rate their cancer care as "*excellent*" or "*very good*". This variation remained after adjusting for clinical factors, such as tumour group and duration of treatment, and trust-level factors. This suggests that the variation by socio-demographic factors is not a result of confounding but is attributable to real differences in experiences among these groups.

To the authors' knowledge, this was the first study to explore cancer patients' overall rating of care by socio-demographic characteristics including longstanding-conditions or disabilities. A principal strength of this study is the large sample size (> 71,000) and the response rate of 68%, which was significantly higher than that achieved by comparable surveys (22,23). Also, as all cancer patients treated by the NHS in England during the assigned 3 month study period were sent the survey, it is likely that findings can be generalised to the wider population of cancer patients. The main limitations of this study relate to the type of data available. The data for the trust quality score was collected approximately three years prior to the NCPES survey period and so may not reflect the quality of the trust at the time of patient admission. The influence of other potentially important predictors of patient experience such as employment status (20), level of deprivation (5) and health status (2,8,24) could not be explored as these data are not gathered through the NCPES. Furthermore, the binary categorisation of patients' responses, as per DH Survey Guidance, condenses the patients' experiences (which were already limited to several multiple choice options) and may mask potentially significant variation.

For peer review only - http://bmjopen.bmj.com/site/about/guidelines.xhtml

Interpretation of the findings from NCPES data requires consideration of the possible determinants of variation in patients' responses to a survey question. Firstly, it is possible that variation reflects differing health, emotional or other support needs that are not met by cancer care services (12,14,25). Secondly, differential expectations between patient groups, perhaps pertaining to socio-cultural norms, may contribute to the observed patterns (8,26). Thirdly, certain patient groups may have a tendency to respond less positively, based on shared norms regarding feedback and ideas as to its purpose (26). Finally, variation may reflect real differences in the quality of care provided (8,26).

Studies of patient experience in the general patient population have demonstrated systematic differences in experience by gender, age and ethnicity and the results of our study further add to this knowledge by demonstrating that similar variation exists among cancer patients. Adjusting for other socio-demographic factors, women were less likely to report positive experiences than men. This may be due to the increased emotional and support needs among female cancer patients described elsewhere (13,14). Breast cancer was the most common tumour group for females (38.4%, n=14,591) and in comparison to other tumour groups breast cancer patients were more likely to rate their care positively. Notably, when clinical factors such as tumour group were adjusted for, the magnitude of the negative association between gender and overall care rating increased. This may indicate that while patient experience varies modestly overall by gender there are marked differences between men and women with less common cancers. This is an area which merits further exploration.

For peer review only - http://bmjopen.bmj.com/site/about/guidelines.xhtml

For peer review only

Younger patients were less likely to report positive experiences than older patients, which corroborates previous findings in relation to age and patient satisfaction (4–8,13, 27). It has been suggested that this observation may reflect a generational phenomenon, whereby older patients' responses are influenced by comparisons with their parents' generation who may not have had access to advanced technologies of modern treatment or the free care provided by the NHS, referred to as 'gratitude bias' (28,29). Alternatively, younger patients may have higher expectations of quality of care due to a reduced frequency of hospital visits compared to older patients (17). The poorer rating of care in the oldest age group (76+ years) fits with neither theory and further work to understand the cause of the variation in cancer patient experience by age is required.

Ethnic minorities, especially Asian and specifically Chinese patients, reported less positive experiences than white patients. This trend is similar to findings from previous studies exploring variation in patient experiences of care generally (2,4,5) and specifically for cancer (17,22). The extent to which these results are due to cultural differences in expectations of care or willingness to criticise is unclear and necessitates further research. Of significant concern is the possibility that these patients experience poorer quality of care owing to a lack of understanding of the care needs of these minority groups or to discrimination, unintended or otherwise (8).

Patients with various long-standing conditions reported significantly less positive patient experiences than those without. The worst experiences were reported by patients with a learning disability or mental health condition. Given the small numbers of patients in these groups and the strength of the association it seems likely that there is marked variation in their experiences compared to other patients. Patients with long-

For peer review only - http://bmjopen.bmj.com/site/about/guidelines.xhtml

standing illnesses such as diabetes and chronic heart disease were also less likely to rate their care as “excellent” or “very good.” Given that the number of patients with such illnesses is set to rise in the future with an ageing patient population it is important to explore how having co-morbidities influences patients’ experience of cancer care.

This study presents evidence of inequalities in experiences of cancer care by gender, age, ethnicity and disability. Whilst it is possible that some of the variation observed between patient groups is a result of varying socio-cultural expectations or tendencies to rate care positively, it is also possible that the quality of care truly differs between patient groups. Further investigation of the experiences of women, ethnic minorities, younger patients and those with a disability is needed so that cancer care services can be better tailored to meet the needs and expectations of these groups. Analysis of the NCPES qualitative free text questions and other patient experience data at a trust level would help to inform quality improvement initiatives. The findings of this study would appear to suggest that, if used as a comparative performance indicator (as is NCPES data) patient experience measures should be adjusted for age, gender and ethnicity. An unadjusted measure of performance could unfairly disadvantage hospital trusts with higher than average proportion of ethnic minority patients, for example. However, the impact of adjusting NCPES data for demographic characteristics on trust rankings has been shown to be minimal (30). Adjusting for gender, age and ethnicity causes few trusts to move into or out of the top or bottom 20% of trusts nationally. While they may not account for much of the between-trust variation in cancer patient experience the overall variation in patient experience by demographic factors is important in its own right and warrants further attention.

For peer review only - http://bmjopen.bmj.com/site/about/guidelines.xhtml

Responses to survey questions are a result of patients' perception and interpretation of events, which are shaped by expectations and clinical or emotional needs, in addition to the quality of services received. Meeting the care needs of all patients equally is a fundamental principle of the NHS and high-quality cancer services must strive to meet the needs of its diverse patient population. This study identifies patient groups for which cancer care services are in greatest need of improvement.

KEY MESSAGES (3-4 sentences less than 25 words each)

- Adjusting for clinical and trust-level factors, there is evidence of inequalities in patients' experiences of cancer care by socio-demographic characteristics.
- Female, younger and non-white (especially Asian) cancer patients are less likely to rate their overall care as excellent or very good.
- Patients with a long-standing condition also report poorer overall care, patients with a learning disability or mental health condition being least satisfied.
- While patient experience varies modestly overall by gender, there may be marked differences between the experiences of men and women with less common cancers.

For peer review only - http://bmjopen.bmj.com/site/about/guidelines.xhtml

LICENCE FOR PUBLICATION STATEMENT

The Corresponding Author has the right to grant on behalf of all authors and does grant on behalf of all authors, an exclusive licence (or non-exclusive for government employees) on a worldwide basis to the BMJ Group and co-owners or contracting owning societies (where published by the BMJ Group on their behalf), and its Licensees to permit this article (if accepted) to be published in Sexually Transmitted Infections and any other BMJ Group products and to exploit all subsidiary rights, as set out in our licence http://group.bmj.com/products/journals/instructions-for-authors/licence-forms.

Acknowledgements: None

Contributorship statement: HW devised the study, advised on data analysis and participated in interpreting the data and reviewing the manuscript; SD participated in interpreting the data and reviewing the manuscript; LM carried out supplementary data analysis, participated in interpreting the data and co-prepared the manuscript; AB carried out the statistical analysis, interpreted the data and co-prepared the manuscript.

Funding: There was no specific funding for this project. HW, SD and LM are supported by the NIHR Imperial College Healthcare NHS Trust Biomedical Research Centre and Imperial College Healthcare Charity.

Competing interests: None

Ethics: No ethical approval was required for this study

Data sharing: The National Cancer Patient Experience Survey 2012-13 dataset used is available for download from http://ukdataservice.ac.uk.

For peer review only - http://bmjopen.bmj.com/site/about/guidelines.xhtml

REFERENCES

1. NHS England. Putting patients first: The NHS England business plan for 2013/14 – 2015/16. 2012 p. 1–64.

2. Healthcare Commission. Variations in the experiences of patients in England: Analysis of the Healthcare Commission's 2003/2004 national surveys of patients. London; 2005 p. 1–35.

3. Department of Health. Report on the self reported experience of patients from black and minority ethnic groups. London; 2009 p. 1–31.

4. Campbell JL, Ramsay J, Green J. Age, gender, socioeconomic, and ethnic differences in patients' assessments of primary health care. *Qual Health Care* 2001;**10**(2):90–5.

5. Commission for Health Improvement. Unpacking the patients' perspective: Variations in NHS patient experience in England. London; 2004 p. 1–112.

6. Sizmur S. Multilevel analysis of inpatient experience. Picker Institute Europe. 2011 p. 1–28.

7. Jenkinson C, Coulter A, Bruster S, et al. Patients' experiences and satisfaction with health care: results of a questionnaire study of specific aspects of care. *Qual Saf Health Care* 2002;**11**(4):335–9.

8. Lyratzopoulos G, Elliott M, Barbiere JM, et al. Understanding ethnic and other socio-demographic differences in patient experience of primary care: evidence

For peer review only - http://bmjopen.bmj.com/site/about/guidelines.xhtml

from the English General Practice Patient Survey. *BMJ Qual Saf* 2012;**21**(1):21–9.

9. Mead N, Roland M. Understanding why some ethnic minority patients evaluate medical care more negatively than white patients: a cross sectional analysis of a routine patient survey in English general practices. *BMJ* 2009;**339**:b3450.

10. Department of Health. Improving Outcomes: A Strategy for Cancer. 2011 p. 1–99.

11. Davidson R, Mills ME. Cancer patients' satisfaction with communication, information and quality of care in a UK region. *Eur J Cancer Care (Engl)* 2005;**14**(1):83–90.

12. Cox A, Jenkins V, Catt S, et al. Information needs and experiences: an audit of UK cancer patients. *Eur J Oncol Nurs* 2006;**10**(4):263–72.

13. Clarke S, Booth L, Velikova G, et al. Social support: gender differences in cancer patients in the United Kingdom. *Cancer Nurs* 2006;**29**(1):66–72.

14. Morrison V, Henderson BJ, Zinovieff F, et al. Common, important, and unmet needs of cancer outpatients. *Eur J Oncol Nurs* 2012;**16**:115–23.

15. Department of Health. Cancer Patient Experience Survey 2011/12: National Report. 2011 p. 1–168.

16. Saunders CL, Abel GA, El Turabi A, et al. Accuracy of routinely recorded ethnic group information compared with self-reported ethnicity: evidence from the English Cancer Patient Experience Survey. *BMJ Open*. 2013;**3**(6):1–9.

For peer review only

For peer review only - http://bmjopen.bmj.com/site/about/guidelines.xhtml

17. El Turabi A, Abel GA, Roland M, et al. Variation in reported experience of involvement in cancer treatment decision making: evidence from the National Cancer Patient Experience Survey. *Br J Cancer* 2013;**109**(3):780–7.

18. Ipsos MORI. Frontiers of performance in the NHS II. 2008 p. 1–100.

19. Raleigh VS, Frosini F, Sizmur S, et al. Do some trusts deliver a consistently better experience for patients? An analysis of patient experience across acute care surveys in English NHS trusts. *BMJ Qual Saf* 2012;**21**(5):381–90.

20. Kontopantelis E, Roland M, Reeves D. Patient experience of access to primary care: identification of predictors in a national patient survey. BMC Fam Pract 2010;**11**(61):1–15.

21. Kreft IG, de Leeuw J. Introducing Multilevel Modeling. London: SAGE Publications; 2002.

22. Lyratzopoulos G, Neal RD, Barbiere JM, et al. Variation in number of general practitioner consultations before hospital referral for cancer: findings from the 2010 National Cancer Patient Experience Survey in England. *Lancet Oncol* 2012;**13**(4):353–65.

23. Care Quality Commission. National NHS patient survey programme: Survey of adult inpatients 2012. 2013 p. 1–34.

24. Cohen G. Age and health status in a patient satisfaction survey. *Soc Sci Med* 1996;**42**(7):1085–93.

25. Pinquart M, Duberstein PR. Information needs and decision-making processes in older cancer patients. *Crit Rev Oncol Hematol* 2004;**51**(1):69–80.

For peer review only - http://bmjopen.bmj.com/site/about/guidelines.xhtml

26. Perneger T. Adjustment for patient characteristics in satisfaction surveys. *Int J Qual Heal Care* 2004;**16**(6):433–5.

27. Crow R, Gage H, Hampson S, et al. The measurement of satisfaction practice from a systematic review. *Health Technol Assess* 2002;**6**(32):1–250.

28. Department of Health. Methods, reasoning and scope: Statement of methodology for the overall patient experience scores. 2013 p. 1–28.

29. Bowling A. An “inverse satisfaction law”? Why don’t older patients criticise health services? *J Epidemiol Community Health* 2002;**56**(7):482.

30. Saunders K, Lyratzopoulos G, Abel G. Is case-mix adjustment important for the National Cancer Patient Experience Survey in England? Cambridge Centre for Health Services Research; 2013 p. 1–12.

For peer review only

For peer review only - http://bmjopen.bmj.com/site/about/guidelines.xhtml

Inequalities in cancer patients' experiences of care: analysis of data from the National Cancer Patient Experience Survey 2011-12

Corresponding author: Louise Mc Grath-Lone, Patient Experience Research Centre, School of Public Health, Imperial College London, W2 1PG, UK.
E-mail:Louise.mc-grath-lone@imperial.ac.uk
Phone: 0207 5943417 Fax: 0207 4023927

Authors: Anna Bone[1], Louise Mc Grath-Lone[2], Sophie Day[2], Helen Ward.[2]

Affiliations:

1. School of Public Health, Imperial College London, W2 1PG, UK.
2. Patient Experience Research Centre, School of Public Health, Imperial College, London, W2 1PG, UK.

Keywords: Patient satisfaction, quality measurement, surveys

Word count: 2,912

For peer review only - http://bmjopen.bmj.com/site/about/guidelines.xhtml

ABSTRACT

Background

Patient experience is a key component of quality in healthcare; however, studies in the UK indicate that patients' experience of care is not equal across gender, age and ethnicity. There are currently 1.8 million people living with cancer in the UK but less is known about their experiences of care. Whether inequalities by socio-demographic characteristics exist amongst cancer patients is not well-documented.

Methods

Using cross-sectional data from 71,793 cancer patients who completed the National Cancer Patient Experience Survey 2011-2012, we examined associations between patient, clinical and trust-level factors and a summary measure of patient experience, namely overall rating of care. Multivariate logistic regression was used to investigate variation by socio-demographic characteristics adjusting for other patient, clinical and trust-level factors.

Results

Female, non-white and younger patients were less likely to rate their overall care as excellent or very good. Patients with long-standing conditions, particularly those with learning disabilities or mental health conditions also reported poorer overall care. This variation persisted when other patient, clinical and trust-level factors were controlled

For peer review only - http://bmjopen.bmj.com/site/about/guidelines.xhtml

for, indicating there are real differences in experiences among cancer patients by socio-demographic characteristics.

Conclusion

There is evidence of inequalities in cancer patient experience in the UK by socio-demographic characteristics such as gender, age, ethnicity and disability. Quality cancer care services must strive to meet the needs of a diverse patient population equally; this study identifies patient groups for whom it appears cancer care services are in greatest need of improvement.

Article summary: Strengths and limitations of this study

- To the authors' knowledge, this is the first study to explore cancer patients' overall rating of care by socio-demographic characteristics including longstanding-conditions or disabilities.
- A principal strength of this study is the large sample size (> 71,000) and high response rate (68%).
- As this study involves secondary analysis of national survey data it is limited by the type of data available e.g. the influence of potentially important predictors of patient experience such as employment status, level of deprivation and health status could not be explored as these data were not gathered.
- A further limitation is that the binary categorisation of patients' responses condenses the patients' experiences (which were already limited to several multiple choice options) and may mask potentially significant variation.

For peer review only - http://bmjopen.bmj.com/site/about/guidelines.xhtml

INTRODUCTION

Patient experience is a key component of quality in healthcare and is one of the top priorities in the NHS (1). However, studies in the UK indicate that there are systematic differences in patient experience by socio-demographic characteristics (2–4). For example, studies on primary and hospital care have found that patients tend to report more positive experiences with increasing age (4–8), females report less positive experiences than males (5,6) and non-white patients report less positive experiences than white patients, even after adjusting for other socio-demographic variables (3,5,6,9). Less is known about variation in the experiences of cancer patients. There are currently 1.8 million people living with cancer in the UK (10) and advances in cancer treatments mean that they are living longer and facing prolonged periods of contact with healthcare services because of complex treatment regimens (11). In 2011, a Department of Health (DH) report set out the government's strategy to improve outcomes by putting patients at the heart of cancer health services (10). A key objective of this strategy was to reduce inequalities in care relating to both clinical outcomes and patient experience. Thus, exploration of the experiences of cancer patients and the inequalities that may exist is critical in order to identify patient groups for whom cancer care services are in greatest need of improvement.

Detailed studies on the experiences of cancer patients have often relied on small sample sizes (12–14). However, a regular National Cancer Patient Experience Survey (NCPES) has been established which provides a wealth of information on care and treatment experiences. The 2011-12 survey includes responses from over 71,000 cancer patients from 160 trusts across the UK (15). With a relatively high response rate and large sample size, the survey presents an opportunity to explore inequalities

For peer review only - http://bmjopen.bmj.com/site/about/guidelines.xhtml

For peer review only

in the quality of care received by cancer patients. Here, we aim to describe the variation in cancer patient experience by age, gender, ethnicity and presence of long-standing conditions or disabilities in order to explore whether there are systematic inequalities. We further examine the influence of clinical and trust-level factors on these variations.

METHODS

Source of data

We carried out a secondary analysis of 2011-2012 NCPES cross-sectional data collected by Quality Health (Chesterfield, UK) on behalf of the DH. All patients with a primary diagnosis of cancer who attended an NHS hospital as an inpatient or day case between 1st September 2011 and 30th November 2011 were sent the survey (15). Non-responders were followed up with two postal reminders. The final response rate achieved was 68%. As no survey weights were available, the data could not be weighted to adjust for non-response. The dataset included demographic and clinical characteristics for 71,793 cancer patients who attended 160 hospital trusts across England, as well as their responses to 70 multiple choice questions relating to various aspects of their experiences of care. Surveys such as NCPES are commonly used to measure patient experience over a range of domains; however, single summary measures of overall experience, such as the Family and Friends Test, have become increasingly important (1). Our analysis focused on a summary measure of patient experience, namely patients' assessment of care as measured by Q70 in the survey, *"Overall, how would you rate your care?"* Responses from a five point scale were transformed into a binary outcome, with "*excellent"* and "*very good"* categorised as

For peer review only - http://bmjopen.bmj.com/site/about/guidelines.xhtml

'positive' and *"good"*, *"fair"* and *"poor"* as 'not positive', in accordance with the DH Survey Guidance (15).

Patient, clinical and trust-level characteristics

The main socio-demographic characteristics of interest in this study (gender, age and ethnicity) were ascertained by self-report (16) and grouped as in the national report (15). As Chinese patients have reported less positive experiences than white patients elsewhere (3,6,17), the "Chinese" ethnic category was not combined with "Other" in this analysis. For age and ethnicity the largest groups were chosen as the reference category. Responses to the question "*Do you have any of the following long-standing conditions?"* were used to identify patients with co-morbidities. The clinical characteristics of tumour group and patient status (i.e. day or inpatient) were taken from hospital administration records.

Haematological cancer patients were assigned as the reference tumour group as the largest group (breast) did not have a representative age and gender distribution. Time since first treatment was ascertained by patients' survey responses. As trust-level factors have previously been associated with patient experience (6,18–20) several were included in this analysis. Hospital trusts were categorised by type (large acute, medium acute, small acute, specialist and teaching) and by foundation status. The Care Quality Commission's (CQCs) 2008/2009 Annual Health Check rating and the proportion of frontline staff satisfied with care at their trust (Q12d from the National NHS Staff Survey 2012) were also included as measures of trusts' overall quality. Quintiles of staff satisfaction were used as a categorical variable during regression

For peer review only - http://bmjopen.bmj.com/site/about/guidelines.xhtml

For peer review only

analysis with the lowest quintile as reference category. The reference categories chosen for other trust-level factors were the largest groups.

Data analysis

Variation in patents' overall rating of care by patient, clinical and trust-level factors was investigated using univariate logistic regression. Respondents with missing demographic, clinical or trust-level data, or those who did not answer Q70, were then excluded (i.e. complete-case analysis was undertaken) and multivariate logistic regression was used to describe associations between the individual demographic characteristics of interest and overall rating of care. Confounding by patient, clinical and trust-level factors was controlled for through their sequential addition to the model. Logistic regression was chosen as the small intra-class coefficient calculated for Q70 (<0.01) suggested the effect of clustering by trust among respondents was negligible; therefore, it was anticipated that a multilevel model and a multivariate logistic regression model would produce similar results (21). However, as even small intra-class correlations can inflate type-1 errors, clustered robust standard errors were utilised. All statistical analyses were conducted in Stata V.12.

For peer review only - http://bmjopen.bmj.com/site/about/guidelines.xhtml

RESULTS

Patient characteristics and rating of care

A total of 71,793 patients admitted to hospital trusts across England with a primary cancer diagnosis completed the survey. Table 1 shows the demographic and clinical characteristics of respondents and Table 2 the characteristics of the hospital trusts they attended. The majority of patients were white, female and >50 years old and there were substantial numbers with disabilities or other long-standing conditions. The most common tumour groups were breast and haematological cancers. Most respondents had started their treatment in the last year and were admitted to hospital as day case patients on their most recent visit. Most were treated in large acute trusts, trusts with foundation status and trusts rated 'Good' by CQC. The majority of patients (96.5%, n=69,276) provided a response to Q70 "*Overall, how would you rate your care?*" In total 87.8% rated their care as "*excellent*" or "*very good*" while the remaining 12.2% rated their care "*good*", "*fair*" or "*poor*" (Fig.1).

For peer review only

Table 1: Characteristics of survey respondents and their unadjusted associations with a positive overall rating of care from univariate logistic regression

Patient characteristics

**Gender**	n	%	OR (95% CI)	*p*-value
Male	33,832	47.1	1 (ref)	
Female	37,961	52.9	0.92 (0.88-0.96)	**<0.001**
**Age group**	n	%	OR (95% CI)	*p*-value
16 - 25	363	0.5	0.68 (0.51-0.92)	**0.01**
26 - 35	969	1.4	0.62 (0.52-0.75)	**<0.001**
36 - 50	6,802	9.5	0.70 (0.64-0.76)	**<0.001**
51 - 65	22,885	31.9	0.79 (0.74-0.83)	**<0.001**
66 - 75	23,643	32.9	1 (ref)	
76+	17,131	23.9	0.85 (0.79-0.90)	**<0.001**
**Ethnicity**	n	%	OR (95% CI)	*p*-value
White	63,652	88.7	1 (ref)	
Mixed	199	0.3	0.66 (0.45-0.97)	**0.04**
Asian/Asian British	1,082	1.5	0.33 (0.29-0.38)	**<0.001**
Black/Black British	885	1.2	0.41 (0.35-0.49)	**<0.001**
Chinese	138	0.2	0.27 (0.19-0.39)	**<0.001**
Other	510	0.7	0.58 (0.46-0.73)	**<0.001**
**Long-standing conditions** [a, b]	n	%	OR (95% CI)	*p*-value
None	48,218	67.2		
Deafness/hearing impairment	7,281	10.1	0.91 (0.85-0.98)	**0.01**
Blindness/partially sighted	1,856	2.6	0.74 (0.65-0.84)	**<0.001**
Physical condition	9,347	13.0	0.71 (0.67-0.76)	**<0.001**
Learning disability	354	0.5	0.50 (0.39-0.65)	**<0.001**
Mental health condition	1,347	1.9	0.55 (0.48-0.64)	**<0.001**
Long-standing illness [c]	9,241	12.9	0.77 (0.73-0.82)	**<0.001**

Clinical characteristics

**Tumour group**	n	%	OR (95% CI)	*p*-value
Brain/CNS	746	1.0	0.52 (0.42-0.63)	**<0.001**
Breast	14,739	20.5	1.00 (0.92-1.09)	0.98
Colorectal / Lower GI	9,483	13.2	0.72 (0.66-0.78)	**<0.001**
Gynaecological	4,202	5.9	0.72 (0.64-0.80)	**<0.001**
Haematological	11,070	15.4	1 (ref)	
Head and Neck	2,422	3.4	0.71 (0.62-0.81)	**<0.001**
Lung	5,029	7.0	0.77 (0.69-0.85)	**<0.001**
Other	1,138	1.6	0.72 (0.59-0.86)	**<0.001**
Prostate	5,831	8.1	0.70 (0.64-0.78)	**<0.001**
Sarcoma	2,451	3.4	0.61 (0.53-0.69)	**<0.001**
Skin	1,695	2.4	0.98 (0.82-1.16)	0.80
Upper GI	4,540	6.3	0.61 (0.55-0.68)	**<0.001**
Urological	8,447	11.8	0.64 (0.58-0.70)	**<0.001**
**Patient status**	n	%	OR (95% CI)	*p*-value
Day case	45,720	63.7	1 (ref)	
Inpatient	26,073	36.3	0.84 (0.80-0.88)	**<0.001**
**Time since first treatment**	n	%	OR (95% CI)	*p*-value
< 1 year	44,997	62.3	1 (ref)	
1-5 years	17,486	24.4	0.83 (0.78-0.87)	**<0.001**
>5 years	6,212	8.7	0.88 (0.81-0.95)	**0.002**

Total number of respondents=71,793. Ethnicity was unknown for 7.4% respondents, long-standing conditions status for 7.3% and time since first treatment for 4.3%. Significant associations at α=0.05 level highlighted in bold.

[a] 6.7% of patients (n=4,780) had >1 long-standing condition, therefore the column total exceeds 100%
[b] Reference category for specific long-standing conditions is not having that condition
[c] Such as (but not limited to) HIV, diabetes, chronic heart disease or epilepsy

For peer review only

Table 2: Characteristics of trusts attended by survey respondents and their unadjusted associations with a positive overall rating of care from univariate logistic regression

Trust-level characteristics				
**Trust type**	n	%	OR (95% CI)	*p*-value
Small acute	6,240	8.7	1.23 (1.12-1.34)	**<0.001**
Medium acute	16,677	23.2	1.07 (1.01-1.14)	**0.02**
Large acute	25,850	36.0	1 (ref)	
Specialist	3,224	4.5	1.54 (1.36-1.76)	**<0.001**
Teaching	19,802	27.6	1.03 (0.98-1.10)	0.24
**Foundation status**	n	%	OR (95% CI)	*p*-value
No	31,798	44.3	0.82 (0.78-0.85)	**<0.001**
Yes	39,995	55.7	1 (ref)	
**CQC trust quality rating (2008/9)**	n	%	OR (95% CI)	*p*-value
Weak	3,926	5.6	0.85 (0.77-0.94)	**0.001**
Fair	18,482	26.2	0.97 (0.92-1.03)	0.34
Good	28,425	40.3	1 ref	
Excellent	19,748	28.0	1.17 (1.10-1.24)	**<0.001**
**Frontline staff satisfied with care** [a]		**Quintiles of frontline staff satisfied with care** [b]		
Mean	63.5%		OR (95% CI)	*p*-value
Median	62.7%	1 (lowest)	1 (ref)	
Range	35.3 - 94.0%	2	1.10 (1.03-1.19)	**0.01**
		3	1.11 (1.03-1.19)	**0.004**
		4	1.17 (1.09-1.26)	**<0.001**
		5 (highest)	1.35 (1.25-1.45)	**<0.001**

Total number of respondents=71,793. CQC trust quality rating was unknown for 3 trusts (1.7% of respondents)and the proportion of frontline staff satisfied with care was unknown for 1 trust (1.7% of respondents).Significant associations at α=0.05 level highlighted in bold.

[a] Calculated from responses to Q12d from the National NHS Staff Survey 2012
[b] Trusts were categorised into quintiles according to the proportion of staff responding positively to Q12d

Abbreviations: CQC=Care Quality Commission

Variation in rating of care by patient, clinical and trust-level factors

Unadjusted associations between patient, clinical and trust-level characteristics and Q70 from univariate logistic regression analysis are shown in Tables 1 & 2. Among all respondents, statistically significant variation in overall rating of care by patient-level characteristics such as ethnicity, gender, age and long-standing conditions was observed. For example, women were less likely than men, and non-white patients were less likely than white patients, to rate their overall care very good or excellent. Chinese patients reported least favourably among non-white ethnic minorities.

For peer review only - http://bmjopen.bmj.com/site/about/guidelines.xhtml

Younger and older patients were less likely than 66-75 year olds to rate their care very good or excellent, with the youngest patients (16-24 year olds) least likely to report excellent or very good overall care. Patients with any long-standing condition were less positive about their overall care; those with a learning disability or mental health condition were the least satisfied.

Clinical and trust-level characteristics were also associated with overall rating of care. With the exception of breast and skin cancer patients, all other patients were less likely than those with haematological cancers to rate their care as very good or excellent. Inpatients, patients who began their treatment more than one year ago and those who attended large acute trusts, trusts without foundation status or trusts with a "weak" CQC rating were also less likely to rate their care as very good or excellent.

Variation in patients' rating of care adjusting for clinical and trust-level factors

After excluding those with missing demographic, clinical or trust-level data, or those who did not provide a rating of their overall care, 60,528 respondents from 150 trusts remained for complete-case analysis. The distribution of patient, clinical and trust-level characteristics in the "complete-case" and "all respondents" populations was similar (Supplemetary Table 1) and there was little difference in the univariate associations between the demographic characteristics and overall rating of care (with the exception of being deaf/having a hearing impairment which was not associated with a poorer rating of overall care during complete-case analysis, Table 3). Model 1 in Table 3 shows the effect of mutually adjusting for all patient-level factors. The observed variation in rating under univariate logistic regression was mostly unaffected; negative

For peer review only - http://bmjopen.bmj.com/site/about/guidelines.xhtml

associations between rating overall care positively and being female, younger, non-white or having a long-standing condition persisted. The magnitude of the associations was generally stable though there was a slight increase in the effect of having a mental health condition or learning disability and being of mixed ethnicity was no longer significantly associated. The addition of clinical factors (tumour group, time since first treatment and in- or day-patient status) to the regression model (Model 2) had little impact on variation by age or ethnicity, but the negative association between being female and care rating increased in magnitude. Including trust-level characteristics in the full multivariate model (Model 3) had a minimal effect on the associations between patients' socio-demographic characteristics and rating of care. Even when adjusting for clinical, trust and other patient-level factors clear variation in patients' rating of care by socio-demographic characteristics such as gender, age, ethnicity and long-standing conditions was evident. Female, younger, non-White patients or patients with a long-standing condition remained less likely to rate their overall care as excellent or very good.

For peer review only

For peer review only - http://bmjopen.bmj.com/site/about/guidelines.xhtml

Table 3: Association between positive rating of overall care and demographic characteristics adjusting for patient, clinical and trust-level factors

	Univariate [a]		Multivariate Model 1 [b]		Multivariate Model 2 [c]		Multivariate Model 3 [d]	
	OR (95% CI)	*p*-value	OR_{adj} (95% CI)	*p*-value	OR_{adj} (95% CI)	*p*-value	OR_{adj} (95% CI)	*p*-value
**Gender**								
Male	1 (ref)		1 (ref)		1 (ref)		1 (ref)	
Female	0.91 (0.87-0.96)	**<0.001**	0.93 (0.88-0.98)	**0.02**	0.72 (0.68-0.76)	**<0.001**	0.72 (0.68-0.77)	**<0.001**
**Age group**								
16 - 25	0.65 (0.48-0.90)	**0.01**	0.68 (0.48-0.98)	**0.04**	0.61 (0.43-0.88)	**0.01**	0.58 (0.41-0.82)	**0.002**
26 - 35	0.67 (0.55-0.81)	**<0.001**	0.71 (0.60-0.86)	**0.001**	0.64 (0.53-0.77)	**<0.001**	0.62 (0.51-0.75)	**<0.001**
36 - 50	0.68 (0.63-0.75)	**<0.001**	0.71 (0.65-0.78)	**<0.001**	0.61 (0.56-0.67)	**<0.001**	0.60 (0.54-0.65)	**<0.001**
51 - 65	0.76 (0.72-0.81)	**<0.001**	0.77 (0.73-0.82)	**<0.001**	0.73 (0.68-0.77)	**<0.001**	0.72 (0.68-0.77)	**<0.001**
66 - 75	1 (ref)		1 (ref)		1 (ref)		1 (ref)	
76+	0.85 (0.79-0.91)	**<0.001**	0.86 (0.80-0.93)	**<0.001**	0.90 (0.83-0.97)	**0.01**	0.90 (0.84-0.98)	**0.01**
**Ethnicity**								
White	1 (ref)		1 (ref)		1 (ref)		1 (ref)	
Mixed	0.65 (0.44-0.97)	**0.04**	0.70 (0.45-1.10)	0.12	0.68 (0.44-1.08)	0.10	0.67 (0.43-1.04)	0.08
Asian	0.35 (0.30-0.40)	**<0.001**	0.37 (0.31-0.44)	**<0.001**	0.35 (0.30-0.42)	**<0.001**	0.35 (0.30-0.41)	**<0.001**
Black	0.45 (0.37-0.53)	**<0.001**	0.46 (0.38-0.56)	**<0.001**	0.46 (0.37-0.56)	**<0.001**	0.43 (0.36-0.53)	**<0.001**
Chinese	0.26 (0.18-0.38)	**<0.001**	0.28 (0.19-0.41)	**<0.001**	0.29 (0.20-0.42)	**<0.001**	0.27 (0.18-0.41)	**<0.001**
Other	0.61 (0.47-0.77)	**<0.001**	0.62 (0.48-0.79)	**<0.001**	0.61 (0.47-0.79)	**<0.001**	0.61 (0.47-0.77)	**<0.001**
**Long-standing conditions [e]**								
Deafness/hearing impairment	0.95 (0.87-1.02)	0.16	0.93 (0.85-0.99)	0.09	0.93 (0.85-1.01)	0.09	0.93 (0.85-1.01)	0.09
Blindness/visual impairment	0.78 (0.68-0.90)	**0.001**	0.85 (0.74-0.97)	**0.01**	0.85 (0.75-0.98)	**<0.001**	0.86 (0.75-0.99)	**0.03**
Physical condition	0.73 (0.68-0.78)	**<0.001**	0.73 (0.69-0.77)	**<0.001**	0.73 (0.69-0.77)	**<0.001**	0.74 (0.70-0.78)	**<0.001**
Learning disability	0.49 (0.38-0.65)	**<0.001**	0.66 (0.49-0.88)	**0.01**	0.68 (0.51-0.90)	**0.01**	0.67 (0.50-0.90)	**0.01**
Mental health condition	0.58 (0.50-0.67)	**<0.001**	0.65 (0.57-0.74)	**<0.001**	0.65 (0.57-0.74)	**<0.001**	0.65 (0.57-0.74)	**<0.001**
Long-standing illness	0.79 (0.74-0.85)	**<0.001**	0.79 (0.74-0.85)	**<0.001**	0.80 (0.75-0.86)	**<0.001**	0.81 (0.75-0.87)	**<0.001**

Significant associations at α=0.05 level with clustered robust standard errors highlighted in bold.

[a] Figures may differ from those presented in Tables 1 & 2 as they are based on 60,528 respondents from 150 trusts with complete data (i.e. complete-case analysis)
[b] Adjusted for patient factors (i.e. ethnicity, gender, age group, specific long-standing conditions)
[c] Adjusted for patient factors and clinical factors (i.e. patient status, tumour group and time since first treatment)
[d] Adjusted for patient factors, clinical factors and trust-level factors (i.e. trust type, foundation status, CQC trust quality rating and quintile of frontline staff satisfied with care)
[e] Reference category for specific long-standing conditions is not having that condition Abbreviations: OR=odds ratio; CI=confidence interval, ref=reference category

For peer review only - http://bmjopen.bmj.com/site/about/guidelines.xhtml

DISCUSSION

Our analysis of the 2011-2012 NCPES demonstrates that there is marked variation in the experiences of cancer patients by socio-demographic factors. Women, younger patients, ethnic minorities and patients with a long-standing condition or disability were less likely to rate their cancer care as "*excellent*" or "*very good*". This variation remained after adjusting for clinical factors, such as tumour group and duration of treatment, and trust-level factors. This suggests that the variation by socio-demographic factors is not a result of confounding but is attributable to real differences in experiences among these groups.

To the authors' knowledge, this was the first study to explore cancer patients' overall rating of care by socio-demographic characteristics including longstanding-conditions or disabilities. A principal strength of this study is the large sample size (> 71,000) and the response rate of 68%, which was significantly higher than that achieved by comparable surveys (22,23). Also, as all cancer patients treated by the NHS in England during the assigned 3 month study period were sent the survey, it is likely that findings can be generalised to the wider population of cancer patients. The main limitations of this study relate to the type of data available. The data for the trust quality score was collected approximately three years prior to the NCPES survey period and so may not reflect the quality of the trust at the time of patient admission. The influence of other potentially important predictors of patient experience such as employment status (20), level of deprivation (5) and health status (2,8,24) could not be explored as these data are not gathered through the NCPES. Furthermore, the binary categorisation of patients' responses, as per DH Survey Guidance, condenses the patients' experiences (which were already limited to several multiple choice options) and may mask potentially significant variation.

For peer review only - http://bmjopen.bmj.com/site/about/guidelines.xhtml

Interpretation of the findings from NCPES data requires consideration of the possible determinants of variation in patients' responses to a survey question. Firstly, it is possible that variation reflects differing health, emotional or other support needs that are not met by cancer care services (12,14,25). Secondly, differential expectations between patient groups, perhaps pertaining to socio-cultural norms, may contribute to the observed patterns (8,26). Thirdly, certain patient groups may have a tendency to respond less positively, based on shared norms regarding feedback and ideas as to its purpose (26). Finally, variation may reflect real differences in the quality of care provided (8,26).

Studies of patient experience in the general patient population have demonstrated systematic differences in experience by gender, age and ethnicity and the results of our study further add to this knowledge by demonstrating that similar variation exists among cancer patients. Adjusting for other socio-demographic factors, women were less likely to report positive experiences than men. This may be due to the increased emotional and support needs among female cancer patients described elsewhere (13,14). Breast cancer was the most common tumour group for females (38.4%, n=14,591) and in comparison to other tumour groups breast cancer patients were more likely to rate their care positively. Notably, when clinical factors such as tumour group were adjusted for, the magnitude of the negative association between gender and overall care rating increased. This may indicate that while patient experience varies modestly overall by gender there are marked differences between men and women with less common cancers. This is an area which merits further exploration.

For peer review only - http://bmjopen.bmj.com/site/about/guidelines.xhtml

For peer review only

Younger patients were less likely to report positive experiences than older patients, which corroborates previous findings in relation to age and patient satisfaction (4–8,13, 27). It has been suggested that this observation may reflect a generational phenomenon, whereby older patients' responses are influenced by comparisons with their parents' generation who may not have had access to advanced technologies of modern treatment or the free care provided by the NHS, referred to as 'gratitude bias' (28,29). Alternatively, younger patients may have higher expectations of quality of care due to a reduced frequency of hospital visits compared to older patients (17). The poorer rating of care in the oldest age group (76+ years) fits with neither theory and further work to understand the cause of the variation in cancer patient experience by age is required.

Ethnic minorities, especially Asian and specifically Chinese patients, reported less positive experiences than white patients. This trend is similar to findings from previous studies exploring variation in patient experiences of care generally (2,4,5) and specifically for cancer (17,22). The extent to which these results are due to cultural differences in expectations of care or willingness to criticise is unclear and necessitates further research. Of significant concern is the possibility that these patients experience poorer quality of care owing to a lack of understanding of the care needs of these minority groups or to discrimination, unintended or otherwise (8).

Patients with various long-standing conditions reported significantly less positive patient experiences than those without. The worst experiences were reported by patients with a learning disability or mental health condition. Given the small numbers of patients in these groups and the strength of the association it seems likely that there is marked variation in their experiences compared to other patients. Patients with long-

For peer review only - http://bmjopen.bmj.com/site/about/guidelines.xhtml

standing illnesses such as diabetes and chronic heart disease were also less likely to rate their care as “excellent” or “very good.” Given that the number of patients with such illnesses is set to rise in the future with an ageing patient population it is important to explore how having co-morbidities influences patients’ experience of cancer care.

This study presents evidence of inequalities in experiences of cancer care by gender, age, ethnicity and disability. Whilst it is possible that some of the variation observed between patient groups is a result of varying socio-cultural expectations or tendencies to rate care positively, it is also possible that the quality of care truly differs between patient groups. Further investigation of the experiences of women, ethnic minorities, younger patients and those with a disability is needed so that cancer care services can be better tailored to meet the needs and expectations of these groups. Analysis of the NCPES qualitative free text questions and other patient experience data at a trust level would help to inform quality improvement initiatives. The findings of this study would appear to suggest that, if used as a comparative performance indicator (as is NCPES data) patient experience measures should be adjusted for age, gender and ethnicity. An unadjusted measure of performance could unfairly disadvantage hospital trusts with higher than average proportion of ethnic minority patients, for example. However, the impact of adjusting NCPES data for demographic characteristics on trust rankings has been shown to be minimal (30). Adjusting for gender, age and ethnicity causes few trusts to move into or out of the top or bottom 20% of trusts nationally. While they may not account for much of the between-trust variation in cancer patient experience the overall variation in patient experience by demographic factors is important in its own right and warrants further attention.

For peer review only - http://bmjopen.bmj.com/site/about/guidelines.xhtml

Responses to survey questions are a result of patients' perception and interpretation of events, which are shaped by expectations and clinical or emotional needs, in addition to the quality of services received. Meeting the care needs of all patients equally is a fundamental principle of the NHS and high-quality cancer services must strive to meet the needs of its diverse patient population. This study identifies patient groups for which cancer care services are in greatest need of improvement.

KEY MESSAGES (3-4 sentences less than 25 words each)

- Adjusting for clinical and trust-level factors, there is evidence of inequalities in patients' experiences of cancer care by socio-demographic characteristics.
- Female, younger and non-white (especially Asian) cancer patients are less likely to rate their overall care as excellent or very good.
- Patients with a long-standing condition also report poorer overall care, patients with a learning disability or mental health condition being least satisfied.
- While patient experience varies modestly overall by gender, there may be marked differences between the experiences of men and women with less common cancers.

For peer review only - http://bmjopen.bmj.com/site/about/guidelines.xhtml

LICENCE FOR PUBLICATION STATEMENT

The Corresponding Author has the right to grant on behalf of all authors and does grant on behalf of all authors, an exclusive licence (or non-exclusive for government employees) on a worldwide basis to the BMJ Group and co-owners or contracting owning societies (where published by the BMJ Group on their behalf), and its Licensees to permit this article (if accepted) to be published in Sexually Transmitted Infections and any other BMJ Group products and to exploit all subsidiary rights, as set out in our licence http://group.bmj.com/products/journals/instructions-for-authors/licence-forms.

Acknowledgements: None

Contributorship statement: HW devised the study, advised on data analysis and participated in interpreting the data and reviewing the manuscript; SD participated in interpreting the data and reviewing the manuscript; LM carried out supplementary data analysis, participated in interpreting the data and co-prepared the manuscript; AB carried out the statistical analysis, interpreted the data and co-prepared the manuscript.

Funding: There was no specific funding for this project. HW, SD and LM are supported by the NIHR Imperial College Healthcare NHS Trust Biomedical Research Centre and Imperial College Healthcare Charity.

Competing interests: None

Ethics: No ethical approval was required for this study

Data sharing: The National Cancer Patient Experience Survey 2012-13 dataset used is available for download from http://ukdataservice.ac.uk.

For peer review only - http://bmjopen.bmj.com/site/about/guidelines.xhtml

For peer review only

REFERENCES

1. NHS England. Putting patients first: The NHS England business plan for 2013/14 – 2015/16. 2012 p. 1–64.

2. Healthcare Commission. Variations in the experiences of patients in England: Analysis of the Healthcare Commission's 2003/2004 national surveys of patients. London; 2005 p. 1–35.

3. Department of Health. Report on the self reported experience of patients from black and minority ethnic groups. London; 2009 p. 1–31.

4. Campbell JL, Ramsay J, Green J. Age, gender, socioeconomic, and ethnic differences in patients' assessments of primary health care. *Qual Health Care* 2001;**10**(2):90–5.

5. Commission for Health Improvement. Unpacking the patients' perspective: Variations in NHS patient experience in England. London; 2004 p. 1–112.

6. Sizmur S. Multilevel analysis of inpatient experience. Picker Institute Europe. 2011 p. 1–28.

7. Jenkinson C, Coulter A, Bruster S, et al. Patients' experiences and satisfaction with health care: results of a questionnaire study of specific aspects of care. *Qual Saf Health Care* 2002;**11**(4):335–9.

8. Lyratzopoulos G, Elliott M, Barbiere JM, et al. Understanding ethnic and other socio-demographic differences in patient experience of primary care: evidence

For peer review only - http://bmjopen.bmj.com/site/about/guidelines.xhtml

from the English General Practice Patient Survey. *BMJ Qual Saf* 2012;**21**(1):21–9.

9. Mead N, Roland M. Understanding why some ethnic minority patients evaluate medical care more negatively than white patients: a cross sectional analysis of a routine patient survey in English general practices. *BMJ* 2009;**339**:b3450.

10. Department of Health. Improving Outcomes: A Strategy for Cancer. 2011 p. 1–99.

11. Davidson R, Mills ME. Cancer patients' satisfaction with communication, information and quality of care in a UK region. *Eur J Cancer Care (Engl)* 2005;**14**(1):83–90.

12. Cox A, Jenkins V, Catt S, et al. Information needs and experiences: an audit of UK cancer patients. *Eur J Oncol Nurs* 2006;**10**(4):263–72.

13. Clarke S, Booth L, Velikova G, et al. Social support: gender differences in cancer patients in the United Kingdom. *Cancer Nurs* 2006;**29**(1):66–72.

14. Morrison V, Henderson BJ, Zinovieff F, et al. Common, important, and unmet needs of cancer outpatients. *Eur J Oncol Nurs* 2012;**16**:115–23.

15. Department of Health. Cancer Patient Experience Survey 2011/12: National Report. 2011 p. 1–168.

16. Saunders CL, Abel GA, El Turabi A, et al. Accuracy of routinely recorded ethnic group information compared with self-reported ethnicity: evidence from the English Cancer Patient Experience Survey. *BMJ Open*. 2013;**3**(6):1–9.

For peer review only - http://bmjopen.bmj.com/site/about/guidelines.xhtml

17. El Turabi A, Abel GA, Roland M, et al. Variation in reported experience of involvement in cancer treatment decision making: evidence from the National Cancer Patient Experience Survey. *Br J Cancer* 2013;**109**(3):780–7.

18. Ipsos MORI. Frontiers of performance in the NHS II. 2008 p. 1–100.

19. Raleigh VS, Frosini F, Sizmur S, et al. Do some trusts deliver a consistently better experience for patients? An analysis of patient experience across acute care surveys in English NHS trusts. *BMJ Qual Saf* 2012;**21**(5):381–90.

20. Kontopantelis E, Roland M, Reeves D. Patient experience of access to primary care: identification of predictors in a national patient survey. BMC Fam Pract 2010;**11**(61):1–15.

21. Kreft IG, de Leeuw J. Introducing Multilevel Modeling. London: SAGE Publications; 2002.

22. Lyratzopoulos G, Neal RD, Barbiere JM, et al. Variation in number of general practitioner consultations before hospital referral for cancer: findings from the 2010 National Cancer Patient Experience Survey in England. *Lancet Oncol* 2012;**13**(4):353–65.

23. Care Quality Commission. National NHS patient survey programme: Survey of adult inpatients 2012. 2013 p. 1–34.

24. Cohen G. Age and health status in a patient satisfaction survey. *Soc Sci Med* 1996;**42**(7):1085–93.

25. Pinquart M, Duberstein PR. Information needs and decision-making processes in older cancer patients. *Crit Rev Oncol Hematol* 2004;**51**(1):69–80.

For peer review only - http://bmjopen.bmj.com/site/about/guidelines.xhtml

26. Perneger T. Adjustment for patient characteristics in satisfaction surveys. *Int J Qual Heal Care* 2004;**16**(6):433–5.

27. Crow R, Gage H, Hampson S, et al. The measurement of satisfaction practice from a systematic review. *Health Technol Assess* 2002;**6**(32):1–250.

28. Department of Health. Methods, reasoning and scope: Statement of methodology for the overall patient experience scores. 2013 p. 1–28.

29. Bowling A. An "inverse satisfaction law"? Why don't older patients criticise health services? *J Epidemiol Community Health* 2002;**56**(7):482.

30. Saunders K, Lyratzopoulos G, Abel G. Is case-mix adjustment important for the National Cancer Patient Experience Survey in England? Cambridge Centre for Health Services Research; 2013 p. 1–12.

For peer review only

For peer review only - http://bmjopen.bmj.com/site/about/guidelines.xhtml

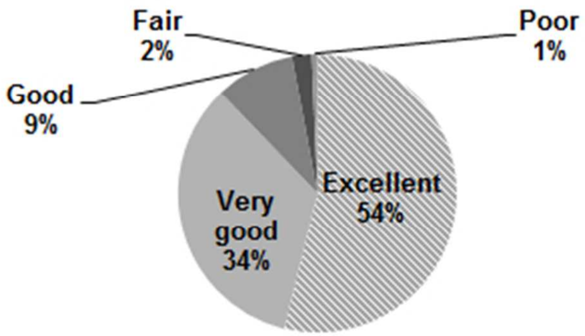

Responses from NCPES 2011-12 to Q70 "Overall, how would you rate your care?"

peer review only

Supplementary table 1: Comparison of the distribution of patient, clinical and trust-level factors in "Case-complete" and "All respondents" populations

		Case-complete	All respondents
Patient factors	***Gender***		
	Male	46.8%	47.1%
	Female	53.2%	52.9%
	Age group		
	16 - 25	0.5%	0.5%
	26 - 35	1.4%	1.4%
	36 - 50	9.7%	9.5%
	51 - 65	32.3%	31.9%
	66 - 75	33.1%	32.9%
	76+	23.1%	23.9%
	Ethnicity		
	White	96.1%	88.7%
	Mixed	0.3%	0.3%
	Asian/Asian British	1.5%	1.5%
	Black/Black British	1.2%	1.2%
	Chinese	0.2%	0.2%
	Other	0.7%	0.7%
	Long-standing conditions		
	None	65.9%	67.2%
	Deafness/hearing impairment	10.5%	10.1%
	Blindness/partially sighted	2.6%	2.6%
	Physical condition	13.7%	13.0%
	Learning disability	0.5%	0.5%
	Mental health condition	1.9%	1.9%
	Long-standing illness [a]	13.5%	12.9%
Clinical factors	***Patient status***		
	Day case	63.8%	63.7%
	Inpatient	36.2%	36.3%
	Time since first treatment		
	< 1 year	64.5%	62.3%
	1-5 years	26.1%	24.4%
	>5 years	9.4%	8.7%
Clinical factors	***Tumour group***		
	Brain/CNS	1.0%	1.0%
	Breast	20.7%	20.5%
	Colorectal / Lower GI	13.2%	13.2%
	Gynaecological	6.0%	5.9%
	Haematological	15.6%	15.4%
	Head and Neck	3.4%	3.4%
	Lung	7.1%	7.0%
	Other	1.6%	1.6%
	Prostate	7.7%	8.1%
	Sarcoma	3.5%	3.4%
	Skin	2.3%	2.4%
	Upper GI	6.3%	6.3%
	Urological	11.7%	11.8%
Trust factors	***Trust type***		
	Small acute	8.6%	8.7%
	Medium acute	22.6%	23.2%
	Large acute	35.8%	36.0%
	Specialist	4.8%	4.5%
	Teaching	28.2%	27.6%
	Foundation status		
	No	42.0%	44.3%
	Yes	58.0%	55.7%
	CQC trust quality rating (2008/9)		
	Weak	5.3%	5.6%
	Fair	26.4 %	26.2%
	Good	40.2%	40.3%
	Excellent	28.1%	28.0%
	Frontline staff satisfied with care [b]		
	Mean	63.1%	63.5%
	Median	63.45	62.7%
	Range	35.3 – 94.0%	35.3 - 94.0%

Number of respondents=60,528 for "Case-complete" population and 71,793 for "All respondents" population.
Significant differences at α=0.05 level highlighted in bold.
[a] Such as (but not limited to) HIV, diabetes, chronic heart disease or epilepsy
[b] Calculated from responses to Q12d from the National NHS Staff Survey 2012

For peer review only

STROBE Statement—Checklist of items that should be included in reports of ***cross-sectional studies***

	Item No	Recommendation		Note
**Title and abstract**	1	(*a*) Indicate the study's design with a commonly used term in the title or the abstract	✓	
		(*b*) Provide in the abstract an informative and balanced summary of what was done and what was found	✓	
**Introduction**				
Background/rationale	2	Explain the scientific background and rationale for the investigation being reported	✓	
Objectives	3	State specific objectives, including any pre-specified hypotheses	✓	
**Methods**				
Study design	4	Present key elements of study design early in the paper	✓	
Setting	5	Describe the setting, locations, and relevant dates, including periods of recruitment, exposure, follow-up, and data collection	✓	
Participants	6	(*a*) Give the eligibility criteria, and the sources and methods of selection of participants	✓	
Variables	7	Clearly define all outcomes, exposures, predictors, potential confounders, and effect modifiers. Give diagnostic criteria, if applicable	✓	
Data sources/ measurement	8*	For each variable of interest, give sources of data and details of methods of assessment (measurement). Describe comparability of assessment methods if there is more than one group	✓	
Bias	9	Describe any efforts to address potential sources of bias	N/A	No weights available
Study size	10	Explain how the study size was arrived at	N/A	Response rate described
Quantitative variables	11	Explain how quantitative variables were handled in the analyses. If applicable, describe which groupings were chosen and why	✓ ✓	
Statistical methods	12	(*a*) Describe all statistical methods, including those used to control for confounding	✓	
		(*b*) Describe any methods used to examine subgroups and interactions	N/A	No sub-analyses done
		(*c*) Explain how missing data were addressed	✓	
		(*d*) If applicable, describe analytical methods taking account of sampling strategy	✓	
		(*e*) Describe any sensitivity analyses	N/A	No sensitivity analyses done
**Results**				
Participants	13*	(a) Report numbers of individuals at each stage of study—eg numbers potentially eligible, examined for eligibility, confirmed eligible, included in the study, completing follow-up, and analysed	✓	

For peer review only - http://bmjopen.bmj.com/site/about/guidelines.xhtml

		(b) Give reasons for non-participation at each stage	N/A	
		(c) Consider use of a flow diagram	N/A	
Descriptive data	14*	(a) Give characteristics of study participants (eg demographic, clinical, social) and information on exposures and potential confounders	✓	
		(b) Indicate number of participants with missing data for each variable of interest	✓	
Outcome data	15*	Report numbers of outcome events or summary measures	✓	
Main results	16	(*a*) Give unadjusted estimates and, if applicable, confounder-adjusted estimates and their precision (eg, 95% confidence interval). Make clear which confounders were adjusted for and why they were included	✓	
		(*b*) Report category boundaries when continuous variables were categorized	✓	
		(*c*) If relevant, consider translating estimates of relative risk into absolute risk for a meaningful time period	N/A	
Other analyses	17	Report other analyses done—eg analyses of subgroups and interactions, and sensitivity analyses	N/A	
**Discussion**				
Key results	18	Summarise key results with reference to study objectives	✓	
Limitations	19	Discuss limitations of the study, taking into account sources of potential bias or imprecision. Discuss both direction and magnitude of any potential bias	✓	
Interpretation	20	Give a cautious overall interpretation of results considering objectives, limitations, multiplicity of analyses, results from similar studies, and other relevant evidence	✓	
Generalisability	21	Discuss the generalisability (external validity) of the study results	✓	
**Other information**				
Funding	22	Give the source of funding and the role of the funders for the present study and, if applicable, for the original study on which the present article is based	✓	

*Give information separately for exposed and unexposed groups.

Note: An Explanation and Elaboration article discusses each checklist item and gives methodological background and published examples of transparent reporting. The STROBE checklist is best used in conjunction with this article (freely available on the Web sites of PLoS Medicine at http://www.plosmedicine.org/, Annals of Internal Medicine at http://www.annals.org/, and Epidemiology at http://www.epidem.com/). Information on the STROBE Initiative is available at www.strobe-statement.org.

For peer review only - http://bmjopen.bmj.com/site/about/guidelines.xhtml
